# Development of a multiplex real-time RT-PCR assay for simultaneous detection of 18 respiratory viruses

**DOI:** 10.3389/fcimb.2025.1727780

**Published:** 2026-01-12

**Authors:** Aijuan Xu, Longxu Xie, Liping Zhong, Xiuli Zhao, Zhenzhou Wan, Jian-Hua Wang, Chiyu Zhang

**Affiliations:** 1 College of Life Sciences, Henan Normal University, Xinxiang, China; 2 Pingyuan Laboratory, Xinxiang, Henan, China; 3 Guangzhou Hybribio Medical Laboratory, Guangzhou, China; 4 Shanghai Public Health Clinical Center, Fudan University, Shanghai, China; 5 Taizhou Fourth People’s Hospital, Taizhou, China

**Keywords:** acute respiratory tract infections, co-infection, primers and probe, multiplex RT-qPCR, specificity, respiratory viruses, human rhinovirus, enterovirus

## Abstract

Acute respiratory tract infections (ARTIs) are a major global health burden with high morbidity and mortality, mainly affecting children under 5 years old and people over 60 years old. The majority of ARTIs are caused by respiratory viruses, such as influenza virus (IFV), respiratory syncytial virus (RSV), human rhinovirus (HRV), human parainfluenza virus (HPIV), human adenovirus (HAdV), human coronavirus (HCoV), human bocavirus (HBoV) and human metapneumovirus (HMPV). Accurate and sensitive diagnosis of respiratory viruses is crucial for clinical treatment and management of patients with ARTIs. Here, we developed a multiplex RT-qPCR assay for detection of 18 common respiratory viruses. The in-house multiplex RT-qPCR assay possesses high sensitivity (limit of detection [LOD]: 5–75 copies/25 μL) and a good linear correlation between the logarithmic copy number and cycle threshold (Ct value, R²≥0.9944), along with excellent specificity characterized by no cross-reactivity within the 18 viruses and no non-specific amplification for other human pathogens. The in-house assay was assessed using 628 clinical samples and compared with Sansure and Easy Diagnosis kits. The results showed a sensitivity of 99.81%, a specificity of 100%, and an overall consistency rate of 99.84% for six target viruses (IAV, IBV, RSV, HRV, HAdV, and SARS-CoV-2). We also reported high prevalences of IAV, HAdV and HRV in Taizhou, Jiangsu, China, and a high potential of HRV co-infection with other respiratory viruses. The new multiplex RT-qPCR assay offers a sensitive, specific, and cost-effective tool for monitoring 18 common respiratory viruses to mitigate the prevalence of ARTIs.

## 1 Introduction

Acute respiratory tract infections (ARTIs) constitute a major global health challenge, marked by high morbidity and mortality rates (Vos et al., 2020). Respiratory viruses are the leading cause of ARTIs with a particular impact on pediatric and geriatric populations (Marrie et al., 1989; Russell et al., 2015). According to the surveillance data from 2009–2019 by the Key Laboratory of Surveillance and Early-Warning on Infectious Diseases, Chinese Center for Disease Control and Prevention, the positive rate of respiratory viruses among children under five years old and school-aged children reached as high as 46.9% (Li et al., 2021). Influenza virus (IFV), respiratory syncytial virus (RSV), human adenovirus (HAdV), human rhinovirus (HRV), and human coronavirus (HCoV) are recognized as the primary etiological agents of ARTIs in the general population. Notably, IFV and RSV infections are responsible for over 300,000 annual deaths worldwide among children under five years of age (Azar et al., 2018). Furthermore, emerging coronaviruses, such as Middle East respiratory syndrome coronavirus (MERS-CoV) and severe acute respiratory syndrome coronavirus 2 (SARS-CoV-2), continue to pose substantial threats to global public health, highlighting the persistent risks associated with this viral genus (Zhang et al., 2020). In particular, co-infections of respiratory viruses with SARS-CoV-2 have been identified as significant contributors to morbidity and mortality in COVID-19 patients, presenting challenges for healthcare providers due to their overlapping clinical manifestations (Ma et al., 2020; Frediansyah et al., 2021; Swets et al., 2022).

Accurate and readily accessible molecular diagnostic techniques for screening and identifying respiratory viruses are crucial to the treatment and management of patients with ARTIs. Traditional diagnostic methods—including cell culture, hemagglutination inhibition tests, enzyme immunoassays, and direct fluorescent antibody assays—are often time-consuming, exhibit low sensitivity or specificity, and are highly dependent on operator expertise (Mahony, 2008; Ginocchio and McAdam, 2011; Vemula et al., 2016). Reverse transcription-quantitative PCR (RT-qPCR) not only offers high sensitivity and specificity but also enables multiplex detection of different pathogens (Mahony, 2010; Zhang et al., 2020; Horemheb-Rubio et al., 2022; Jiang et al., 2022). Therefore, RT-qPCR technology has been considered as a gold standard method for molecular diagnosis of pathogens globally.

In this study, we developed a multiplex RT-qPCR assay for detection of 18 common respiratory viruses, including influenza A virus (IAV), influenza B virus (IBV), influenza C virus (ICV), RSV, HRV, enterovirus (EV), HAdV, SARS-CoV-2, human coronavirus HKU1 (HCoV-HKU1), human coronavirus 229E (HCoV-229E), human coronavirus NL63 (HCoV-NL63), human coronavirus OC43 (HCoV-OC43), human metapneumovirus (HMPV), human bocavirus (HBoV), human parainfluenza virus type 1 (HPIV-1), HPIV-2, HPIV-3, and HPIV-4. The in-house multiplex RT-qPCR assay demonstrates high sensitivity with a limit of detection (LOD) ranging from 5–75 copies/25 μL, and exhibits excellent specificity with no cross-reactivity observed among 18 non-target viruses. Clinical evaluation using 628 clinical samples showed that the in-house multiplex RT-qPCR assay exhibited a sensitivity of 99.81%, a specificity of 100% and an overall consistency rate of 99.84% for the six target viruses (IAV, IBV, RSV, HRV, HAdV and SARS-CoV-2) when compared with commercially available kits.

## 2 Materials and methods

### 2.1 Design of primers and probes

To obtain optimal primers and probes for detection of respiratory viruses, 2–7 sets of primers and probes were designed for each virus using Oligo7 software (http://oligo.net) or retrieved from previous papers (**Supplementary Table S1**) (Sanghavi et al., 2011; Lee et al., 2021; Mancini et al., 2021; Yüce et al., 2021). The primers and probe were specifically tailored by selecting the most conserved genomic regions of the viruses. The specificities of primers and probes were preliminarily verified using Primer-BLAST (https://www.ncbi.nlm.nih.gov/tools/primer-blast/index.cgi?LINKLOC=BlastHome). To evaluate the adaptations to virus variants or quasispecies, the primers and probes were analyzed by aligning with corresponding viral sequences downloaded from GenBank (**Supplementary Figure S1**). The human B2M gene was selected as the internal control (IC) for the detection of 18 respiratory viruses. The probes were designed by incorporating fluorescent dyes (CY5/FAM/VIC/ROX) and quenchers (BHQ1/BHQ2) at the 5’ and 3’ ends, respectively. All primers and probes were synthesized by General Biotechnology Co., Ltd. (Anhui, China) and reconstituted in molecular-grade RNase-free water (Vazyme, China).

### 2.2 Preparation of RNA standards

Plasmids PUC-57 containing the target gene sequences of IAV, IBV, ICV, RSV, HRV, EV, HAdV, SARS-CoV-2, HCoV-HKU1, HCoV-229E, HCoV-NL63, HCoV-OC43, HMPV, HBoV, HPIV-1, HPIV-2, HPIV-3 and HPIV-4 were synthesized by General Biol (Anhui, China). For RNA viruses, their target gene fragments were amplified from the plasmids using specific PCR primers and Taq DNA Polymerase (Vazyme, China) following the manufacturer’s instructions. A T7 promoter was added to the 5’ end of the forward or reverse PCR primer according to the genomic strand of the virus (**Supplementary Table S2**). PCR amplicons were separated by agarose gel electrophoresis and purified using the FastPure Gel DNA Extraction Mini Kit (Vazyme, China). After quantification with a NanoDrop2000 spectrophotometer (Thermo Fisher Scientific, USA), 0.1-0.5 µg of the purified PCR products were used as templates for *in vitro* transcription with the T7 High Yield RNA Transcription Kit (Vazyme, China) at 37°C for approximately 16 hours. The newly synthesized RNA was treated with DNase I at 37°C for 15 minutes. RNA concentration was measured using a NanoDrop2000, and the RNA was diluted in TE solution (Sigma, USA). RNase inhibitor (Vazyme, China) was added to the RNA storage solution, which was then aliquoted and stored at -80°C. The copy number of RNA standards was calculated using the formula: RNA copies per mL = [RNA concentration (ng/mL)/(transcript length in nt × 340)] × 6.022 × 10²³ and the copy number of DNA standards was calculated using the formula: DNA copies per mL = [DNA concentration (ng/mL)/(transcript length in nt × 660)] × 6.022 × 10²³.

### 2.3 Establishment of singleplex and multiplex RT-qPCR assays

The singleplex RT-qPCR reaction system consists of 0.5× amplification buffer, 200 μM dNTPs, 1 U Taq DNA polymerase, 2 U reverse transcriptase, 20 U RNase inhibitor (Vazyme, China), 200 nM of each primer, and 100 nM probe. The multiplex RT-qPCR reaction system includes 1× amplification buffer, 200 μM dNTPs, 2 U Taq DNA polymerase, 40 U reverse transcriptase, 30 U RNase inhibitor, 0.15 U UNG enzyme (Vazyme, China), and the primers and probe for three viruses. 3 μL DNA/RNA standards or samples were added in each reaction. The thermal cycling conditions were as follows: reverse transcription at 55°C for 15 min; enzyme activation and initial denaturation at 95°C for 30 s; followed by 45 cycles of denaturation at 95°C for 10 s and combined annealing/extension at 60°C for 1 min. Fluorescence signals were collected during the annealing step using an ABI 7500 PCR Detection System (Thermo Fisher Scientific, USA).

### 2.4 Screening for optimal primer sets

To screen the optimal primer and probe set for each virus, parallel experiments were performed with the same reaction system and conditions except different sets of primers and probes. The primer and probe set yielding the lowest cycle threshold (Ct) value was selected for the RT-qPCR assay (**Supplementary Figure S2**). To improve the performance, some probes were modified by incorporating minor groove binder (MGB) to shorten the probe length.

### 2.5 Standard curves for quantification and limit of detection

The standard curves of the multiplex RT-qPCR assays were generated using 10-fold serial dilutions of RNA or DNA standards ranging from 10^6^ to 10^0^ copies/μL. The LODs of the multiplex assays were determined using 10-fold or 5-fold serial dilutions of RNA or DNA standards. Each dilution was tested with 20 replicates. The LOD was defined as a 95% probability of obtaining a positive result, using probit regression analysis with SPSS 26.0 software.

### 2.6 Specificity of the multiple RT-qPCR assays

**Figure 1:**
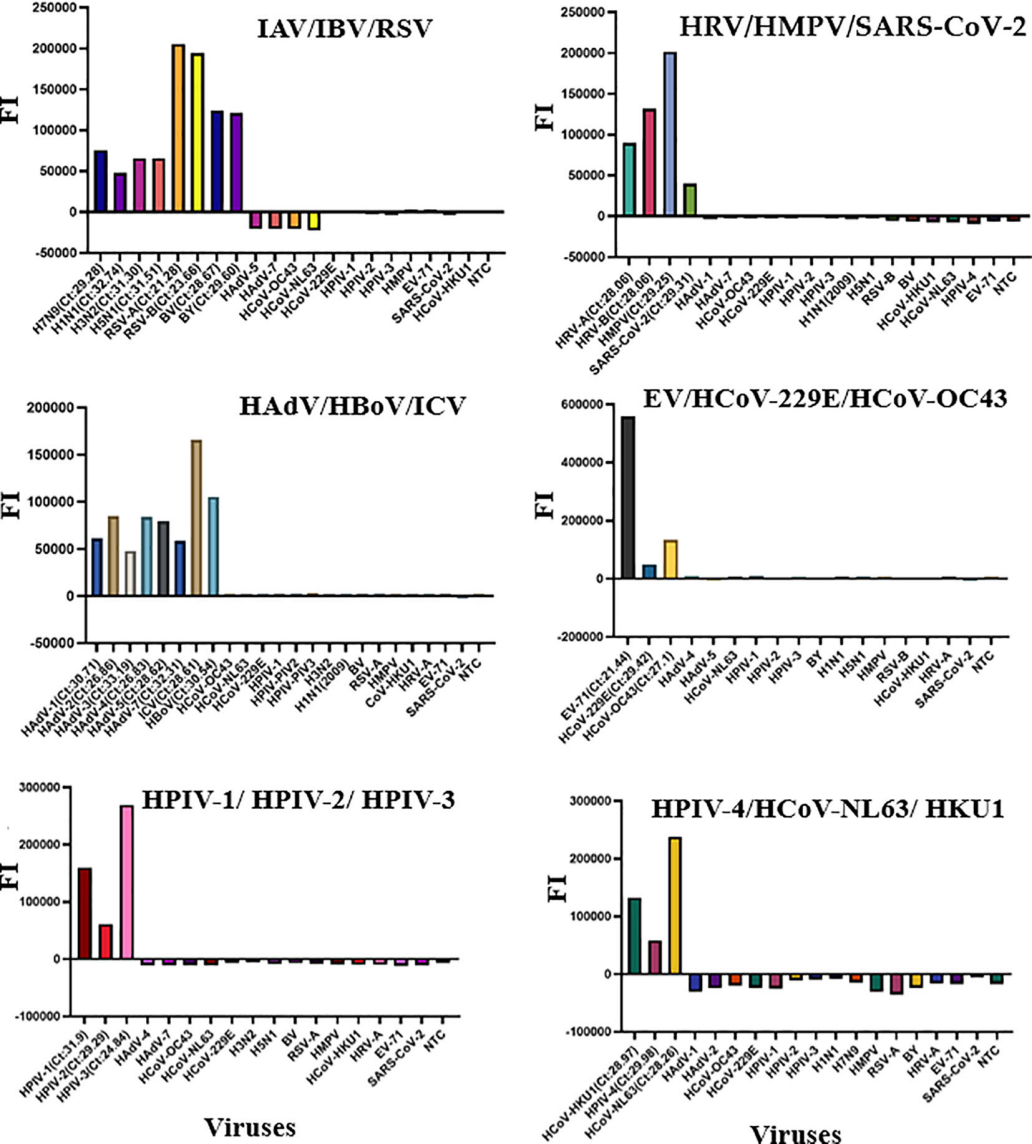
The specificity of the multiplex RT-qPCR assay. *FI*, fluorescence intensity.

As mentioned above, the specificities of primers and probes were firstly assessed by against all available genomic sequences of the 18 respiratory viruses in GenBank with Primer-BLAST (https://www.ncbi.nlm.nih.gov/tools/primer-blast/index.cgi?LINKLOC=BlastHome). Then, the specificity of the multiplex assay was evaluated using 24 virus isolates and 1 clinical sample previously confirmed positive for HBoV. The virus isolates were purchased from BeNa Culture Collection (BNCC) (Zhengzhou, China), including IAV-H1N1 (2009), IAV-H5N1, IAV-H3N2, IAV-H7N9, IBV-Yamagata (BY), IBV-Victoria (BV), RSV-A, RSV-B, HRV-A, HRV-B, EV-71, HAdV-1, HAdV-2, HAdV-3, HAdV-4, HAdV-5, HAdV-7, SARS-CoV-2, HCoV-229E, HCoV-OC43, HMPV, HPIV-1, HPIV-2 and HPIV-3. All these virus isolates have concentration of 10^4^ copies/mL, except RSV-A and RSV-B, both of which had a concentration of 10^5^ copies/mL. The HBoV-positive sample was determined to have a concentration of 2.14×10^5^ copies/mL. These virus isolates and clinical sample had Ct values of 21–32 in the RT-qPCR assays (**Figure 1**). Because of lack of virus isolates and positive clinical samples for HPIV-4, HCoV-NL63, HCoV-HKU1 and ICV, we used four recombinant plasmids containing target gene fragments to evaluate analytical specificity of the assays and their concentrations were all 10^6^ copies/mL. The target sequences were retrieved from GenBank (accession numbers: e.g., HPIV-4: ON778025.1; HCoV-NL63: LC687395.1; HCoV-HKU1: NC_006577.2; ICV: AB000611.1) and synthesized into pUC57 vectors by General Bio (Anhui) Co., Ltd.

### 2.7 Intra-assay and inter-assay reproducibility of the multiplex RT-qPCR assays

To evaluate the stability of the assays, intra-assay tests were respectively performed with 10 replicates at three different days. The intra-batch and inter-batch mean values, standard deviations, and coefficients of variation were calculated.

### 2.8 Evaluation with clinical samples

A total of 628 oropharyngeal swab samples were collected from outpatients, inpatients (with community-acquired pneumonia, upper respiratory tract infections, and other related diseases), and healthy individuals at Taizhou Fourth People’s Hospital during March 2024 to March 2025.

Viral DNA/RNA was extracted from 300 μL of each oral swab sample using a DNA/RNA extraction kit (Tianlong Biotech Inc., Shanxi Province, China) and eluted in 60 μL of nuclease-free water. The extracted DNA/RNA was stored at −80°C until use. 5 μL of DNA/RNA sample was added to the reaction.

A parallel test was performed using commercial RT-qPCR assays (Sansure Biotech Inc. and EasyDiagnosis) for each sample. The commercial kit from Sansure Biotech Inc. (Hunan Province) can detect influenza A virus, influenza B virus, RSV, HRV, HAdV, and Mycoplasma pneumoniae. The kit from EasyDiagnosis (Hubei Province) was used to detect SARS-CoV-2.

To validate the discrepant results between our in-house multiplex assay and the commercial kits, two complementary validation strategies were employed: first, nested RT-PCR assays were developed and conducted (details in **Supplementary Table S3**), with their amplification products targeting pathogens that are also detected by the commercial kits; second, specific primers for Sanger sequencing were designed, which targeted pathogens uniquely included in our in-house multiplex assay panel. For pathogens showing inconsistent detection results between the two platforms, Sanger sequencing was performed using an ABI 3730 Genetic Analyzer (Thermo Fisher Scientific, USA) to confirm their presence or absence. All sequences obtained from this validation process are provided in **Supplementary Table S4**.

### 2.9 Statistical analysis

The performance of the multiplex RT-qPCR assay was assessed by sensitivity (SE), specificity (SP) and consistency rate (CR). SE is calculated by SE = TP/(TP + FN) × 100%. SP is calculated by SP = TN/(FP +TN) × 100%. CR is calculated by CR = (TP + TN)/(TP + TN + FP + FN). A Kappa test was performed with SPSS v 26.0 software to estimate the degree of consistency between our multiplex RT-qPCR assays and the commercial RT-qPCR kits. The Kappa statistic is interpreted as follows: <0, as poor; 0–0.20, as slight; 0.21–0.40, as fair; 0.41–0.60, as moderate; 0.61–0.80, as substantial; and 0.81–1.00, as almost perfect agreement (Liu et al., 2014).

## 3 Results

### 3.1 Optimization of the multiple RT-qPCR assay

The reaction with the earliest amplification curve (i.e., the lowest Ct value) indicates the highest amplification efficiency. Two to seven sets of primers and probes were subjected to the screening of the optimal primer/probe set. Based on amplification efficiency and specificity, the primer/probe set with the lowest Ct value and without non-specific amplification (no amplification curve in non-template control) was selected for each virus. As a result, IAV-set5, IBV-set1, RSV-set5, HRV-set2, SARS-CoV-2-set1, HMPV-set1, ICV-set1, HBoV-set5, HAdV-set7, HCoV-OC43-set3, HCoV-229E-set2, EV-set3, HCoV-HKU1-set1, HCoV-NL63-set2, HPIV-4-set1, HPIV-1-set5, HPIV-2-set4, and HPIV-3-set3 were selected for the establishment of the singleplex and multiplex RT-qPCR assays since they exhibited the highest amplification efficiency and well specificity (**Supplementary Figure S2**). To cover 18 respiratory viruses, six independent triplex reactions (tubes) were established, including tube 1: IAV, IBV and RSV; tube2: HRV, SARS-CoV-2 and HMPV; tube3: ICV, HBoV and HAdV; tube4: EV, HCoV-229E and HCoV-OC43; tube5: HPIV-1, HPIV-2 and HPIV-3; tube6: HCoV-NL63, HCoV-HKU1 and HPIV-4 (**Table 1**). The optimal reaction mixture of each multiplex reaction was obtained by further optimizing the amounts of Taq DNA polymerase, RNA inhibitor, RT, UNG, as well as the concentrations of dNTPs, primers and probes (**Supplementary Figure S3**).

**Table 1:** The information of the primers and probes used in the multiple RT-qPCR assay.

Reaction	Viruses	Primers/probes	Sequences (5’-3’)’	Target gene	Ref
Tube 1	IAV	FRP	GTCTTCTAACCGAGGTCGAAACGAGGTGACAGGATYGGTCTTGTCTTVIC-CRTCYGGCCCCCTCAAAGCCGA-BHQ1	M	Thisstudy
	IBV	FRP	TCCTCAACTCACTCTTCGAGCGCGGTGCTCTTGACCAAATTGGROX-CCAATTCGAGCAGCTGAAACTGCGGT-BHQ2	NS&NEP	(Mancini et al., 2021)
	RSV	FRP	GGGCAAATATGGAAACATACGGCACCCATATTGTTAGTGATGCFAM-CTTCACGARGGCTCCACATACACAG-3'BHQ2+TMP	M	(Lee et al., 2021)
Tube 2	HRV	FRP	TGTGAAGASYCGCGTGTGCGTCCCATCCCRCAATTRCTCFAM-TCCTCCGGCCCCTGAATGCGGCTAA-BHQ1	5’UTR	This study
	SARS-CoV-2	FRP	TTCTCCTGCTAGAATGGCTGGCATTTTGCTCTCAAGCTGGTTCAATCVIC-TGATGCTGCTCTTGCTTTGCTGCTGCT-BHQ1	N	This study
	HMPV	FRP	TGGVAAAGCWTTAGGCTCATCTGTTAGATGAYCTGGCRATGACROX-CCCCACCTYAGCATTGTTTGACCAGCWC-BHQ2	N	This study
Tube 3	ICV	FRP	CTGGAGACTTYTTGGGAGTGGAGTTCTCAACCAAGCTGTGATTGTTCCROX-ACCCAGACGACTACACACCAGACATCCG-BHQ2	M	This study
	HBoV	FRP	CCTATATAAGCCGATGCACTTCGCTTTTTCCAGAGATGTTCACVIC-TCAGACTGCATCCGGTCTC-BHQ1	NS1&NP1	This study
	HAdV	FRP	CCAAAGGMCCCCATCCARGTCTACTTYCAYCACATCAACAGFAM-ATYGGCTCKCATCCTCGCAC- 3'MGB+TMP	Hexon	This study
Tube 4	HCoV-OC43	FRP	TCAATACCCCGGCTGACATCCTGAGCCTTCAATATAGTAACCCFAM-TRCCAGGCGGAAACCTAGTCGGAAT-BHQ1	N&M	This study
	HCoV-229E	FRP	GTACCCCTAAGCCTTCTCGTAATTAGGCTGTCTTTTCCACCGTVIC-AGTCCTGCTTCTTCTCAATCTGCTGCC-BHQ1	N	This study
	EV	FRP	GCAASTCTGCRGCGGAACCATTGTCACCATAAGCAGCCAROX-CTACTTTGGGTGTCCGTGTTTCCT-BHQ2	5’UTR	This study
Tube 5	HPIV-1	FRP	CAACAGGAAATCATGTTCTGTAATAGCAAATACTAAGTCTTCTATACCTTCACFAM-AGTTATGCTCCTTGCCCACTGTGAATG-BHQ1	HN	This study
	HPIV-2	FRP	GSRTTTCCAATCTTCAGGACTATGCAAGTCTCAGTTCAGCTAGRTCVIC-AATCAATCGSAAAAGCTGTTCAGTCAC-BHQ1	HN	This study
	HPIV-3	FRP	TCTCGGGTATGGAGGTCTTGAACAATGCYTGATTRCAGTCTCTCTGTGTROX-CCAGGTCACCCAGTTGTGTTGCAGA-BHQ2	HN	This study
Tube 6	HCoV-HKU1	FRP	GCGGAGGAGTTGGCTAGTAATGCAGCGACAATCTATCTTCAROX-ATGCCCCTCTACTGGTCAAGCGATGG-BHQ1	ORF 1ab	This study
	HCoV-NL63	FRP	TAGTGGTAAGTCACATTGTTCCATCAATGCTATAAACAGTCATAGCTTFAM-CTTGTGCCCATGCTGCTGTTGATTCCT-BHQ1	ORF 1ab	This study
	HPIV-4	FRP	AGATCACAATGAACTACWGATGCCAYTCTGATGGGTGGAKTCTGGTGVIC-TTCTGGGATGAGTGGTGYTCTGRCTGT-BHQ1	N	This study

The adenine (A) residue marked in red is modified with the TMP probe, aiming to enhance detection sensitivity and accelerate the reaction.

### 3.2 Specificity for the RT-qPCR assays

A total of 29 pathogens, including 24 culture strains (IAV-H1N1 (2009), IAV-H5N1, IAV-H3N2, IAV-H7N9, IBV-Yama (BY), IBV-Victoria (BV), RSV-A, RSV-B, HRV-A, HRV-B, EV-71, HAdV-1, HAdV-2, HAdV-3, HAdV-4, HAdV-5, HAdV-7, SARS-CoV-2, HCoV-OC43, HCoV-229E, HMPV, HPIV-1, HPIV-2, HPIV-3), 1 clinical sample positive for HBoV and 4 Plasmids (HPIV-4, HCoV-NL63, HCoV-HKU1 and ICV) were used to determine the specificity of the multiplex RT-qPCR in this study. Neither non-specific amplification nor cross-reaction was observed among these pathogens, indicating high clinical and analytical specificity of the RT-qPCR assays (**Figure 1**, **Supplementary Figure S4**).

### 3.3 LOD for RT-qPCR assay

The LODs of the single RT-qPCR assay for IAV, IBV, ICV, RSV, HRV, EV, HAdV, SARS-CoV-2, HCoV-HKU1, HCoV-229E, HCoV-NL63, HCoV-OC43, HMPV, HPIV-1, HPIV-2, HPIV-3, HPIV-4 and HBoV were determined as 8, 44, 75, 17, 21, 50, 68, 9, 16, 25, 39, 15, 36, 17, 40, 30, 14 and 152 copies per 25 μL reaction, respectively (**Table 2**). The LODs of the multiple-RT-qPCR assays for IAV, IBV, ICV, RSV, HRV, EV, HAdV, SARS-CoV-2, HCoV-HKU1, HCoV-229E, HCoV-NL63, HCoV-OC43, HMPV, HPIV-1, HPIV-2, HPIV-3, HPIV-4 and HBoV were 5, 50, 18, 11, 38, 50, 75, 11, 17, 15, 54, 15, 31, 7, 31, 71, 9 and 41 copies per 25 μL reaction, respectively (**Table 2**).

**Table 2:** LOD of the multiple RT-qPCR assay.

Panels	Viruses	LOD (copies/25 µL reaction)	
		Multiplex reaction	Singleplex reaction
Tube 1	IAV	5	8
	IBV	50	44
	RSV	11	17
Tube 2	HRV	38	21
	SARS-CoV-2	11	9
	HMPV	31	36
Tube 3	ICV	18	75
	HBoV	41	152
	HAdV	75	68
Tube 4	HCoV-OC43	13	15
	HCoV-229E	15	25
	EV	50	50
Tube 5	HPIV-1	7	17
	HPIV-2	31	40
	HPIV-3	71	30
Tube 6	HCoV-HKU1	17	16
	HCoV-NL63	54	39
	HPIV-4	9	14

### 3.4 Standard curves for the RT-qPCR assays

**Figure 2:**
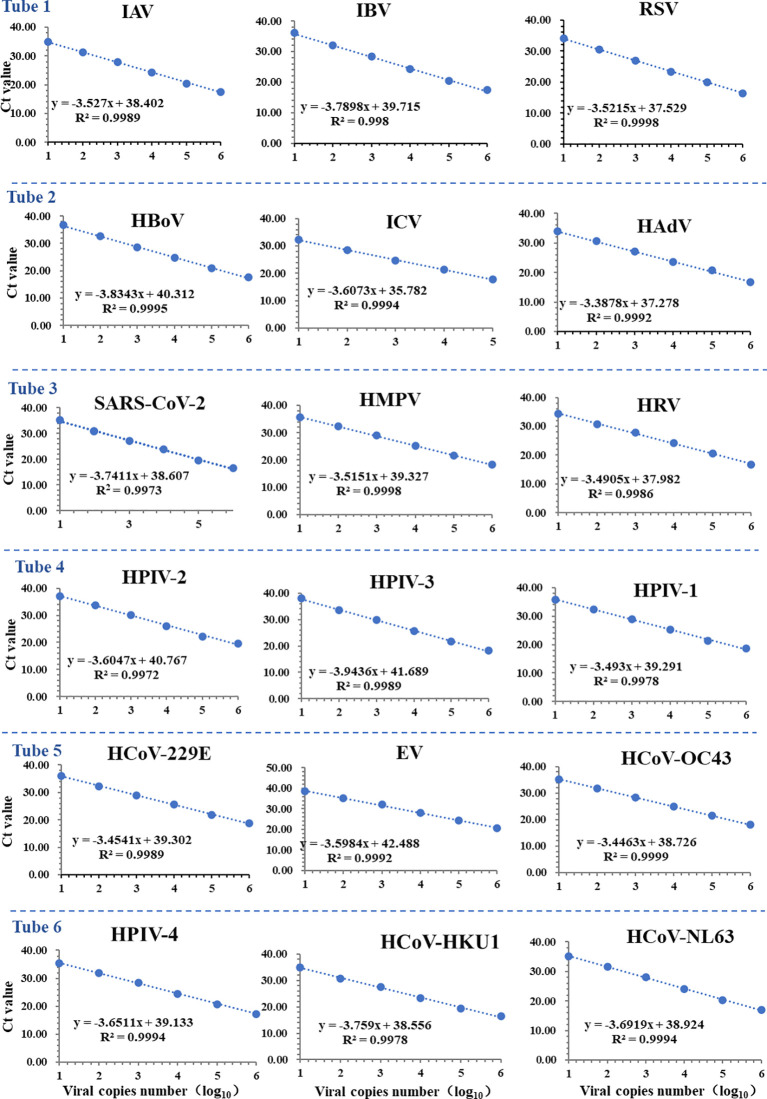
Standard curves of the multiplex RT-qPCR assay for 18 respiratory viruses. There was a good linear correlation between the logarithmic copy number and cycle threshold (Ct value) for each virus (R² ≥ 0.9972).

A good linear relationship between the log copy number and the Ct value in the standard curve was observed for each virus within the scope from 1 × 10^1^ to 1 × 10^6^ copies/μL, regardless of the singleplex (**Supplementary Figure S5**) and multiplex RT-qPCR assays (**Figure 2**). The correlation coefficients (R^2^) of the singleplex RT-qPCR assays for IAV, IBV, ICV, RSV, HRV, EV, HAdV, SARS-CoV-2, HCoV-HKU1, HCoV-229E, HCoV-NL63, HCoV-OC43, HMPV, HPIV-1, HPIV-2, HPIV-3, HPIV-4 and HBoV were 0.9988, 0.9987, 0.9993, 0.997, 0.9994, 0.9988, 0.9971, 0.9944, 0.9995, 0.9965, 0.9989, 0.9986, 0.9998, 0.9997, 0.9976, 0.9996, 0.9993 and 0.9993, respectively. TheR^2^ of the multiplex RT-qPCR assays for IAV, IBV, ICV, RSV, HRV, EV, HAdV, SARS-CoV-2, HCoV-HKU1, HCoV-229E, HCoV-NL63, HCoV-OC43, HMPV, HPIV-1, HPIV-2, HPIV-3, HPIV-4 and HBoV were 0.9989, 0.998, 0.9994, 0.9998, 0.9986, 0.9992, 0.9992, 0.9973, 0.9978, 0.9989, 0.9994, 0.9999, 0.9998, 0.9978, 0.9972, 0.9989, 0.9994 and 0.9995, respectively. These results indicate that the assays can be well used for absolute quantification of viral load.

### 3.5 Intra-assay and inter-assay reproducibility of the multiplex RT-qPCR assays

The intra-assay reproducibility of the multiplex RT-qPCR assays were assessed using 10 replicates of 3000 copies template input per 25 μL reaction. Very small intra coefficient of variations of 0.18-1.68% were observed for the multiplex RT-qPCR assays for the 18 respiratory viruses (**Table 3**). Similarly, inter coefficient of variation was 0.36-1.71%. These results indicate good consistency in the detection performance of the multiplex assays (**Table 3**).

**Table 3:** Intra-assay and inter-assay reproducibility tests of the multiplex RT-qPCR assay.

Panels	Concentration of standard plasmids (3000copies/25 µL reaction)	Intra-coefficient of variation			Inter-coefficient of variation		
		n	Mean ± SD	CV%	n	Mean ± SD	CV%
Tube 1	IAV	10	27.72 ± 0.47	1.68%	3	27.33 ± 0.47	1.71%
	IBV	10	27.33 ± 0.12	0.43%	3	27.33 ± 0.12	0.45%
	RSV	10	20.27 ± 0.11	0.56%	3	20.17 ± 0.13	0.64%
Tube 2	HRV	10	26.35 ± 0.15	0.56%	3	26.37 ± 0.13	0.49%
	HMPV	10	27.63 ± 0.11	0.40%	3	27.66 ± 0.10	0.36%
	SARS-CoV-2	10	28.73 ± 0.15	0.51%	3	28.94 ± 0.20	0.68%
Tube 3	HAdV	10	27.99 ± 0.15	0.54%	3	27.9 ± 0.14	0.52%
	HBoV	10	30.36 ± 0.16	0.54%	3	30.41 ± 0.15	0.50%
	ICV	10	27.18 ± 0.11	0.42%	3	26.72 ± 0.36	1.34%
Tube 4	EV	10	24.64 ± 0.14	0.58%	3	24.7 ± 0.17	0.67%
	HCoV-OC43	10	28.35 ± 0.34	1.19%	3	28.71 ± 0.38	1.33%
	HCoV-29E	10	28.49 ± 0.15	0.53%	3	28.52 ± 0.18	0.62%
Tube 5	HPIV-1	10	26.76 ± 0.30	1.13%	3	26.89 ± 0.26	0.98%
	HPIV-2	10	29.68 ± 0.25	0.85%	3	30.13 ± 0.42	1.38%
	HPIV-3	10	28.57 ± 0.16	0.55%	3	28.51 ± 0.14	0.50%
Tube 6	HCoV-HKU1	10	27.8 ± 0.11	0.40%	3	27.47 ± 0.29	1.06%
	HCoV-NL63	10	27.52 ± 0.33	1.18%	3	27.45 ± 0.30	1.10%
	HPIV-4	10	26.4 ± 0.05	0.18%	3	26.32 ± 0.10	0.39%

### 3.6 Clinical performance of the new in-house multiplex RT-qPCR assay

**Figure 3:**
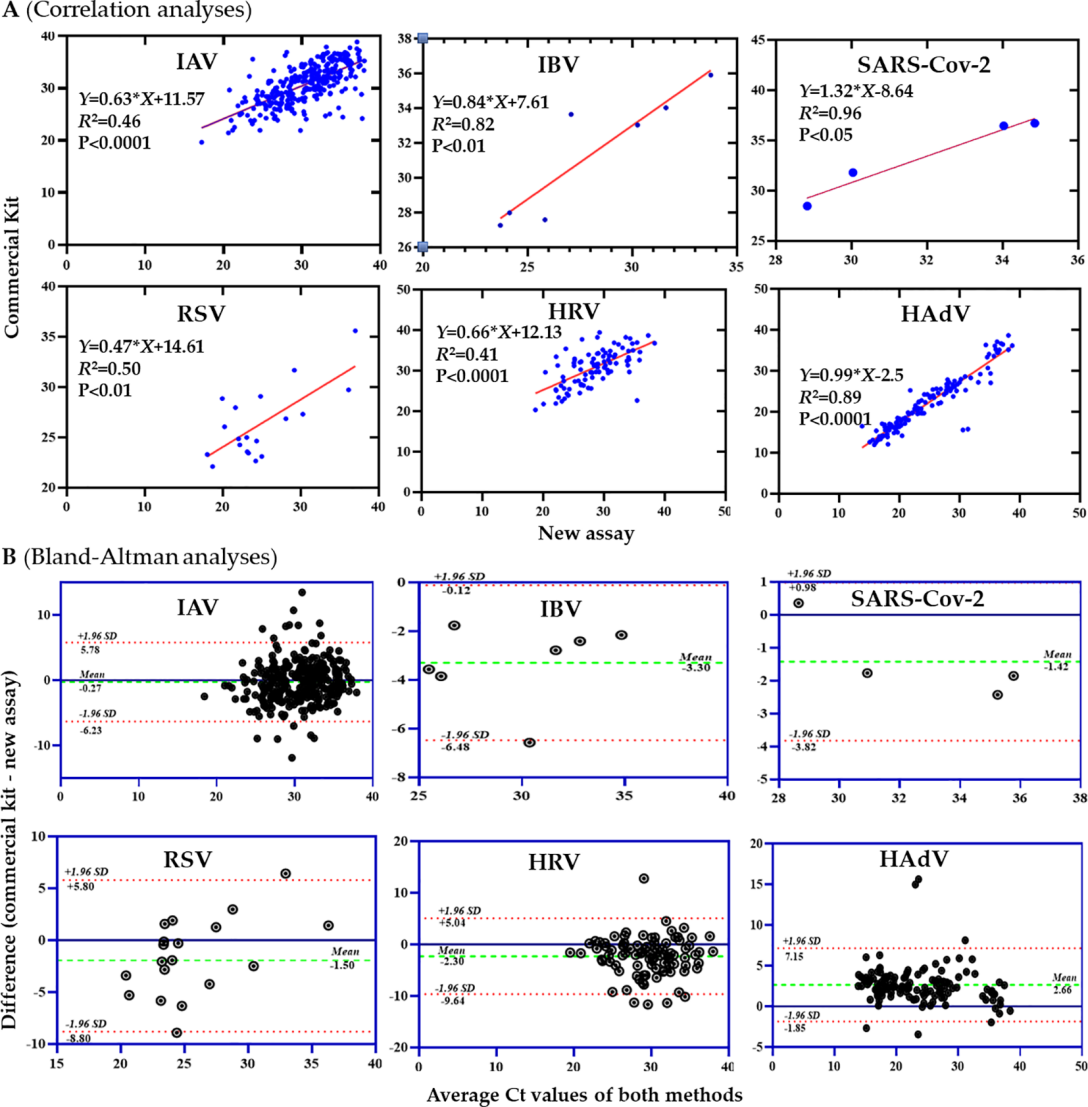
Comparison of the new multiplex RT-qPCR assay with commercial kits for six respiratory viruses. **(A)** Ct value scatter plots. **(B)** Bland-Altman analyses.

To evaluate the clinical performance of the multiplex RT-qPCR assays, we performed a comparative analysis of the new multiplex RT-qPCR assays with the commercial kits in detection of six respiratory viruses (IAV, IBV, RSV, HRV, HAdV, and SARS-Cov-2) using 628 oropharyngeal swab samples. Relevant information regarding the specimens included in this study is summarized in **Table 4**. Furthermore, the Ct values of IBV, HAdV, and SARS-CoV-2 detected by the in-house RT-qPCR assay showed a significant linear correlation with those obtained using commercial RT-qPCR kits, with R² values of 0.82, 0.89, and 0.92, respectively (all P-values < 0.05; specifically, P < 0.01, P < 0.0001, and P < 0.05) (**Figure 3**).

**Table 4:** Socio-demographics information of 628 patients for screening of respiratory viruses.

Patients (n=628)	
Age, years (Mean ± SD)	35.6 ± 32.8
Range of age (years old)	2月-98岁
<5	109 (17.4%)
5-18	217(34.7%)
19-35	18 (2.9%)
36-60	68 (10.8%)
>60	216 (34.4%)
Gender	
Male	332(52.9%)
Female	296 (47.1%)
Diseases	
Upper Respiratory Tract Infection	195 (31.0%)
Respiratory Failure and Dyspnea	8 (1.3%)
Acute Bronchitis, Tonsillitis	115 (18.0%)
Non-severe Community-Acquired Pneumonia, Pulmonary Infection	189 (30.1%)
Severe Community-Acquired Pneumonia Complicated with Acute Exacerbation of Chronic Obstructive Pulmonary Disease	26(4.1%)
Healthy Individuals	6 (1.0%)
Other (Not Related to Lung Infection)	89 (14.2%)

Notably, the primary clinical manifestations of the patients from whom these specimens were collected included symptoms associated with upper respiratory tract infection (URTI) and pneumonia. Of these samples, 532 (84.7%) samples were detected as positive for any one of the six viruses by the new in-house multiplex RT-qPCR assay and the commercial kits (**Table 5**). Both methods had consistent detection rates for RSV (3.1%), IBV (1.1%) and SARS-CoV-2 (0.6%). The commercial kit detected 309 (49.2%) and 92 (14.6%) positives for IAV and HRV, respectively, with one more positive than the new in-house method for each virus. One IAV-positive sample only by the commercial kit had a Ct value of 38, and failed to be confirmed by a nested RT-PCR assay, suggesting a very low viral load. One HRV-positive sample only by the commercial kit was detected positive to EV and further confirmed to EVA71 by Sanger sequencing (**Supplementary Table S4**). We performed in-silico cross-reactivity analysis by aligning HRV-specific primers and probe with 18 representative strains of five enteroviruses, including 4 EVA71, 4 EVD68, 3 Coxsackievirus A16 (CVA16), 4 CVA6, and 3 echovirus 30 (accession numbers listed in **Supplementary Table S5**). No significant homologous sequences to the HRV-primers and probe were found in the genomes of the enteroviruses, supporting high specificity of the in-house assay and low possibility of cross-reactivity to enteroviruses. The in-house method detected 138 positive samples for HAdV, obviously more than 132 by the commercial kit. All 6 HAdV-positive samples only by the in-house method were confirmed by Sanger sequencing. Then we determined true positive and true negative samples for each virus based on the consistent detection results by both the in-house assay and commercial kits, and confirmation results for inconsistent samples experiments by Sanger sequencing, and then assessed the performance of the in-house method. The results showed that the new in-house method exhibited 100% specificity and sensitivity in detection of IAV, IBV, RSV, HAdV and SARS-CoV-2 (**Table 6**). The new in-house assay demonstrated a better performance for HADV and HRV than the commercial kit, but a slightly lower sensitivity for IAV than the commercial kit.Co-infection of respiratory viruses are frequent in patients with ARTIs. The in-house multiplex assay detected 33 (5.3%) dual infections and 2 triplex infections among above six respiratory viruses, respectively, more than 30 (4.8%) and 1 (0.16%) by the commercial kits (**Table 5**). These results indicate that new in-house method demonstrated have better performance than the commercial RT-qPCR kits in detection of respiratory viruses especially for the detection of coinfections.

**Table 5:** Detection of common respiratory viruses in 628 clinical samples by the new in-house and commercial RT-qPCR assays.

Infection pattern	Viruses	New multiplex RT-qPCR assays		Commercial RT-qPCR kits	
		Pos.(n)	PR(%)	Pos.(n)	PR(%)
Overall	IAV	308	49.0%	309#	49.2%
	IBV	7	1.1%	7	1.1%
	RSV	19	3.0%	19	3.0%
	HRV	91	14.5%	92*	14.6%
	HAdV	138	22.0%	132	21.0%
	SARS-Cov-2	4	0.6%	4	0.6%
	Sum 1: Any one of six viruses	532	84.7%	532	84.7%
	HPIV-1	4	0.6%	NA	NA
	HPIV-2	1	0.2%	NA	NA
	HPIV-3	2	0.3%	NA	NA
	HPIV-4	1	0.2%	NA	NA
	EV	4	0.6%	NA	NA
	HCoV-229E	1	0.2%	NA	NA
	HBoV	1	0.2%	NA	NA
	HMPV	1	0.2%	NA	NA
	Sum 2: Any one of above 14 respiratory viruses	535	85.2%	NA	NA
Dual infection	IAV、HRV	16	2.6%	17¶	2.7%
	HAdV、HRV	7	1.1%	6	1.0%
	IAV、HAdV	4	0.6%	3	0.5%
	HAdV、RSV	2	0.3%	1	0.2%
	HRV、RSV	1	0.2%	1	0.2%
	IAV、RSV	1	0.2%	1	0.2%
	Sum 3: any one of above dual infection patterns	33	5.3%	30	4.8%
	HAdV、HPIV-1	1	0.2%	NA	NA
	HAdV、HPIV-4	1	0.2%	NA	NA
	HRV、EV	3	0.5%	NA	NA
	IAV、HPIV-1	2	0.3%	NA	NA
	IAV、HPIV-3	1	0.2%	NA	NA
	RSV、HBoV	1	0.2%	NA	NA
	IAV、HCoV-229E	1	0.2%	NA	NA
	Sum 4: all dual infection patterns	41	6.5%	NA	NA
Triplex infection	IAV、HAdV、HRV	2	0.3%	1	0.2%
	IAV、HAdV、HMPV	1	0.2%	NA	NA
	Total	3	0.5%		

#The Ct value by the commercial kit was 38, a value very close to the cutoff of positive. *One HRV positive sample identified by the commercial kit was detected positive for EV by the new in-house RT-qPCR assay and further confirmed as EVA71 by Sanger sequencing and blast search (**Supplementary Table S4**). One dual infection with IAV and HRV by the commercial kit was detected as triplex infection with IAV, HRV and HAdV by the new in-house RT-qPCR assay with confirmation of HAdV coinfection by Sanger sequencing and blast search. NA, not applicable. Pos., positive. PR, prevalence rate.

**Table 6:** Comparison between the commercial RT-qPCR kits and the new multiplex RT-qPCR assay.

Viruses	Methods	TP	TN	FP	FN	Se (%)	Sp (%)	ACC (%)
IAV	Commercial kit	309	319	0	0	100%	100%	100%
	In-house	308	320	0	1	99.7%	100%	99.8%
IBV	Commercial kit	7	621	0	0	100%	100%	100%
	In-house	7	621	0	0	100%	100%	100%
RSV	Commercial kit	19	609	0	0	100%	100%	100%
	In-house	19	609	0	0	100%	100%	100%
HRV	Commercial kit	91	536	1	0	100%	99.8%	99.8%
	In-house	91	537	0	0	100%	100%	100%
HAdV	Commercial kit	132	490	0	6	95.6%	100%	99.0%
	In-house	138	490	0	0	100%	100%	100%
SARS-CoV-2	Commercial kit	4	624	0	0	100%	100%	100%
	In-house	4	624	0	0	100%	100%	100%

TN, true negative; FP, false positive; FN, false negative; Se, sensitivity; Sp, specificity; Acc, accuracy.

Apart from above 6 respiratory viruses, the new in-house RT-qPCR assay also detected 15 (2.4%) positive for other respiratory viruses, including 4 HPIV-1, 1 HPIV-2, 2 HPIV-3, 1 HPIV-4, 4 EV, 1 HCoV-229E, 1 HBoV, and 1 HMPV. Of 15 positive samples, 11 were coinfected with one or two of IAV, HAdV, HRV, and RSV.

In summary, 14 respiratory viruses were detected among 536 (85.4%) of 628 oropharyngeal samples. IAV was the predominant respiratory virus (49.2%) circulating in this cohort, followed by HAdV (22.0%) and HRV (14.5%). It was not surprising that the top three dominant respiratory viruses were involved in almost all 44 coinfection events except one dual infection by RSV and HBoV, including IAV in 27 (61.4%), HADV in18 (40.9%) and HRV in 29 (65.9%). Furthermore, we found that HRV (29/91, 31.9%) had higher possibility of coinfecting with other respiratory viruses than HAdV (18/138, 13.0%) and IAV (27/309, 8.7%).

## 4 Discussion

In this study, we developed an in-house multiplex RT-qPCR assay for detection of 18 common respiratory viruses, encompassing influenza A/B/C viruses (IAV/IBV/ICV), RSV, HRV, EV, HAdV, SARS-CoV-2, four human coronaviruses (HCoV-HKU1, HCoV-229E, HCoV-NL63, and HCoV-OC43), HMPV, HBoV, and four human parainfluenza viruses (HPIV-1/2/3/4). The multiplex assay demonstrated high analytical sensitivity with LOD of 5–75 copies/25 μL reaction, great linear relationship (R² ≥ 0.9944) between log copy numbers and Ct values, and high analytical reproducibility with intra-assay CV of 0.18–1.68%, and inter-assay CV of 0.36–1.71%. No cross-reactivity among these 18 viruses and no non-specific amplification for other human pathogens indicate good specificity. Clinical evaluation with 628 clinical samples showed a better performance of the in-house multiplex RT-qPCR assay than the commercial kits at least for IAV, IBV, RSV, HRV, HAdV and SARS-CoV-2.

qPCR is widely recognized as the gold standard for molecular diagnosis due to its high sensitivity, specificity, and capacity to enable multiplex detection. Developments of multiplex qPCR assay for various pathogens play a crucial role in the prevention and control of infectious diseases. An inherent challenge for the development of a multiplex qPCR assay is mutual interference and non-specific hybridization among the primers and probes targeting different pathogens, which may reduce detection sensitivity and cause cross-reactivity, therefore leading to false results. In this study, we implemented three strategies to address these issues. First, 2–7 sets of primers and probes were designed for each virus and then subjected to preliminary screening experiments to obtain the primer and probe set with the highest amplification efficiency and high specificity with no non-specificity amplification in non-template control. Second, we performed sequence alignment to assess the identity of each primer and probe to the viral target using all available viral sequences from GenBank. To improve the coverage to highly heterogeneous viral sequences, degenerate bases were incorporated to corresponding primers and probes (e.g. the primers for HRV and HAdV) to minimize mismatches with viral variants. Furthermore, AutoDimer was used to evaluate the thermodynamic stability of the primers and probes and Hetero-Dimer Analysis was performed to exclude the non-specific hybridization among the primers and probes. Third, minor groove binder (MGB) was used to improve the hybridization affinity and specificity of probe (e.g. the probe for HAdV). These strategies enabled the same detection sensitivity (almost the same LOD) and specificity of the multiplex RT-qPCR assays to the singleplex assays.

The majority of ARTIs are caused by a diverse array of respiratory viruses, many of which have multiple genotypes or subtypes. For instance, HRV comprise over 150 subtypes, and HMPV has two major genotypes (A, B). The in-house multiplex RT-qPCR assay covers 18 common respiratory viruses, and show good specificity to detect and distinguish these viruses. Both HRV and EV belong to the family *Picornaviridae*, and share high genetic similarity especially at the 5’ untranslated region (5’UTR) of their genomes. As the relatively conserved region in viral genome, 5’-UTRs of HRV and EV are often used as a target genomic region for designing primers and probe, thus resulting in cross-amplification between both viruses (Ng et al., 2025). Of 628 clinical samples, one EV-positive sample was misclassified as positive for HRV by the commercial assay, but was correctly identified by the in-house assay and further confirmed by Sanger sequencing, indicating the in-house RT-qPCR assay has high specificity to detect and distinguish these respiratory viruses.

Coinfections with two or more respiratory viruses are often observed in children with ARTIs, and are associated with more severe outcomes, including increased hospitalization duration, respiratory failure, and mortality (Le Glass et al., 2021; Adams et al., 2022; Abo et al., 2023; Cao et al., 2024; de Hoog et al., 2025). In this study, we detected 44 (7.0%) coinfection cases, including 41 dual infections and 3 triple infections. The most commonly observed coinfection pattern was dual infections with IAV and HRV (16 cases, 2.6%), followed by dual infections with HAdV and HRV (7 cases, 1.1%) and IAV and HAdV (4 cases, 0.6%). A multicenter study in China (Li et al., 2024) reported that IAV and HRV co-infection was associated with a 2.3-fold higher risk of severe pneumonia in adults, likely due to synergistic immune dysregulation. HRV infections can induce epithelial damage that facilitates IAV entry, and the latter can suppress type I interferon responses, promoting HRV replication (Di Maio et al., 2025). Furthermore, HRV (29/91, 31.9%) appeared to have a very high possibility of coinfecting with other respiratory viruses. Most respiratory viral infections cause mild and self-limiting diseases. HRV co-infections with other respiratory viruses (e.g. HCoV-229E) can aggravate the disease (Madewell et al., 2023), such as increased cough severity, longer symptom duration (≥7 days), and higher rates of hospital admission in children and older adults. Therefore, surveillance of HRV coinfection is very important for clinical treatment and management of patients with ARTIs especially in the spring and winter seasons when influenza and other respiratory viruses are prevalent. Compared with commercial RT-qPCR kits, the in-house multiplex RT-qPCR assay demonstrates comparable detection sensitivity, but offers broader coverage of respiratory viruses, lower assay costs, and superior capability for identifying respiratory virus coinfections (**Supplementary Table S6**). Therefore, the new in-house multiplex RT-qPCR assay can be used as a robust tool for surveillance of respiratory viral infections.

This study has three limitations. First, the in-house RT-qPCR assay focuses exclusively on respiratory viruses and does not cover bacterial and atypical bacterial etiologies of ARTIs (e.g. *Streptococcus pneumoniae*, *Mycoplasma pneumoniae*, *Chlamydia pneumoniae*). *S. pneumoniae* and *M. pneumoniae* are the top bacterial causes of pneumonia in China, and their co-infection with respiratory viruses (e.g. IAV) can exacerbate disease severity. The RT-qPCR assay can be updated to contain these bacterial pathogens in future. Second, IAV, HAdV and HRV are among the top respiratory viruses circulating in China. However, the three viruses are separately tested in three independent reactions. A new combination pattern containing the three viruses in a single reaction may facilitate the monitoring of these respiratory viruses. Third, the clinical performance of the in-house RT-qPCR assay was assessed only for six respiratory viruses (i.e., SARS-CoV-2, IAV, IBV, RSV, HRV, and HAdV), but not for other respiratory viruses. The main reason is lack of commercial kits approved by the National Medical Products Administration (NMPA) for other respiratory viruses. Furthermore, analytical specificity of the new assay to HPIV-4, HCoV-NL63, HCoV-NL63, and ICV was determined by plasmids containing target gene fragments, rather than full-length genome due to lack of virus isolates and clinical samples positive for these viruses. Experiments should be performed to further determine the specificity and clinical performance of the new assay in future.

In conclusion, the in-house multiplex RT-qPCR assay offers a sensitive, specific, and comprehensive tool for detecting 18 common respiratory viruses. Its key strengths include high detection sensitivity (LOD: 5–75 copies per 25 μL reaction), specificity, and reproducibility, broad coverage of respiratory viruses, and excellent performance in clinical samples. Furthermore, we reported high prevalences of IAV, HAdV and HRV in Taizhou, Jiangsu, China, and a high potential of HRV coinfection with other respiratory viruses.

## Data Availability

The original contributions presented in the study are included in the article/**Supplementary Material**. Further inquiries can be directed to the corresponding authors.
