# Supplementary material for: Development of a multiplex real-time RT-PCR assay for simultaneous detection of 18 respiratory viruses
Source: Front Cell Infect Microbiol. 2026 Jan 12;15:1727780. doi: 10.3389/fcimb.2025.1727780 (PMC12833288; doi:10.3389/fcimb.2025.1727780)
Supplement: Supplementary file 1 [file Table1.docx]

Supplementary Material

**Supplementary Table S1.** **All RT-qPCR primer sets used in the screening experiments.**

| **Viruses** | **Primer sets** | **Primer name** | **Primer sequence (5'-3')** | **Target Region** | **Source** |
| --- | --- | --- | --- | --- | --- |
| IAV | IAV-set1 | Probe | VIC-TGCAGTCCTCGCTCACTGGGCACG  -BHQ1 | M | (Mancini et al., 2021) |
|  |  | Forward | GACCAATCCTGTCACCTCTGAC |  |  |
|  |  | Reverse | AGGGCATTYTGRACAAAGCGTCTA |  |  |
|  | IAV-set2 | Probe | VIC-TGCAGTCCTCGCTCACTGGGCACG  -BHQ1 |  | This study |
|  |  | Forward | AGACAAGACCAATCYTGTCACC |  |  |
|  |  | Reverse | AAACCCATTYCCATTDAGGGCATT |  |  |
|  | IAV-set3 | Probe | VIC-CTCACCGTGCCCAGTGAGC-BHQ1 |  | (Lee et al., 2021) |
|  |  | Forward | ACAAGACCAATCCTGTCACCT |  |  |
|  |  | Reverse | TGGACAAAGCGTCTACGCT |  |  |
|  | IAV-set4 | Probe | VIC-CTCACCGTGCCCAGTGAGC-BHQ1 |  | This study |
|  |  | Forward | ACAAGACCAATCCTGTCACCT |  |  |
|  |  | Reverse | TGGACAAAGCGTCTACGCT |  |  |
|  | IAV-set5 | Probe | VIC-CRTCYGGCCCCCTCAAAGCCGA-BHQ1 |  | This study |
|  |  | Forward | GTCTTCTAACCGAGGTCGAAAC |  |  |
|  |  | Reverse | GAGGTGACAGGATYGGTCTTGTCTT |  |  |
| IBV | IBV-set1 | Probe | ROX-CCAATTCGAGCAGCTGAAACTGC  GGT-BHQ2 | NS&  NEP | (Mancini et al., 2021) |
|  |  | Forward | TCCTCAACTCACTCTTCGAGCG |  |  |
|  |  | Reverse | CGGTGCTCTTGACCAAATTGG |  |  |
|  | IBV-set2 | Probe | ROX-AGCAACAAGCCTTCCACTCTGGT  C-BHQ2 |  | This study |
|  |  | Forward | ACCCCAATGGATACAAGTCCT |  |  |
|  |  | Reverse | GCCATCTTCTTCATCCTCCACT |  |  |
| RSV | RSV-set1 | Probe | FAM-CATCCAGCAAATACAC-MGB | N | This study |
|  |  | Forward | TGATACACTSAACAAAGATCAACTTCTG |  |  |
|  |  | Reverse | TCTCCTGTGCTMCGTTGRAT |  |  |
|  | RSV-set2 | Probe | FAM-TAATAGGTATGTTATATGCKATGT  C-BHQ1 |  | (Sanghavi et al., 2011) |
|  |  | Forward | CACWGAAGATGCWAATCATAAATTCA |  |  |
|  |  | Reverse | GTATYTTTATRGTGTCTTCYCTTCCTAACC |  |  |
|  | RSV-set3 | Probe | FAM-CTTCACGAAGGCTCCACATACA  CAGCWG-BHQ1 | M | This study |
|  |  | Forward | GCAAATATGGAAACATACGTGAACA |  |  |
|  |  | Reverse | CATCGTCTTTTTCTAGGACATTGTA |  |  |
|  | RSV-set4 | Probe | FAM-CTTCACGAAGGCTCCACATACAC  AGCAG-BHQ1 |  | This study |
|  |  | Forward | GCAAATATGGAAACATACGTGAACA |  |  |
|  |  | Reverse | CATCGTCTTTTTCTAGGACATTGTA |  |  |
|  | RSV-set5 | Probe | FAM-CTTCACGARGGCTCCACATACA  CAG-BHQ2+TMP |  | (Lee et al., 2021) |
|  |  | Forward | GGGCAAATATGGAAACATACG |  |  |
|  |  | Reverse | GCACCCATATTGTTAGTGATGC |  |  |
| HRV | HRV-set1 | Probe | FAM-TCCTCCGGCCCCTGAATGCGGC  TAA-BHQ1 | 5’UTR | This study |
|  |  | Forward | TGGACARGGTGTGAAGAGC |  |  |
|  |  | Reverse | CAAAGTAGTYGGTCCCATCC |  |  |
|  | HRV-set2 | Probe | FAM-TCCTCCGGCCCCTGAATGCGGC  TAA-BHQ1 |  |  |
|  |  | Forward | TGTGAAGASYCGCGTGTGC |  |  |
|  |  | Reverse | GTCCCATCCCRCAATTRCTC |  |  |
|  | HRV-set3 | Probe | FAM-AGCCRCATTCAGGGGCCG-BHQ1 |  |  |
|  |  | Forward | GACARGGTGKGAAGRKYC |  |  |
|  |  | Reverse | ACACGGACACCCAAAGTAGT |  |  |
| SARS-CoV-2 | SARS-CoV-2-set1 | Probe | VIC-TGATGCTGCTCTTGCTTTGCTGCT  GCT-BHQ1 | N | This study |
|  |  | Forward | TTCTCCTGCTAGAATGGCTGGC |  |  |
|  |  | Reverse | ATTTTGCTCTCAAGCTGGTTCAATC |  |  |
|  | SARS-CoV-2-set2 | Probe | VIC-ACTTCCTCAAGGAACAACATTG  CCA-BHQ1 |  | (Frediansyah et al., 2021) |
|  |  | Forward | CACATTGGCACCCGCAATC |  |  |
|  |  | Reverse | GAGGAACGAGAAGAGGCTTG |  |  |
|  | SARS-CoV-2-set3 | Probe | VIC-CAGGTGGAACCTCATCAGGAGA  TGC-BHQ1 | RdRP | (Frediansyah et al., 2021) |
|  |  | Forward | GTGARATGGTCATGTGTGGCGG |  |  |
|  |  | Reverse | CARATGTTAAASACACTATTAGCATA |  |  |
| HMPV | HMPV-set1 | Probe | ROX-CCCCACCTYAGCATTGTTTGACCA  GCWC-BHQ2 | N | This study |
|  |  | Forward | TGGVAAAGCWTTAGGCTCATC |  |  |
|  |  | Reverse | TGTTAGATGAYCTGGCRATGAC |  |  |
|  | HMPV-set2 | Probe | ROX-TCCCACAAAATCAGAGRCCTTCA  GCACCAG-BHQ2 |  | This study |
|  |  | Forward | AGACAAAGARGCAAGAAARACAATGG |  |  |
|  |  | Reverse | GCTAGTTTRGTGAATATTAAGGCACC |  |  |
|  | HMPV-set3 | Probe | ROX-TGYAATGATGAGGGTGTCACTGC  GGTTG-BHQ2 |  | (Feng et al., 2018) |
|  |  | Forward | CATATAAGCATGCTATATTAAAAGAGTCTC |  |  |
|  |  | Reverse | CCTATTTCTGCAGCATATTTGTAATCAG |  |  |
|  | HMPV-set4 | Probe | ROX-TGYAATGATGAGGGTGTCACTGC  GGTTG-BHQ2 |  | This study |
|  |  | Forward | ACACAATAAARAGAGATGTAGGCAC |  |  |
|  |  | Reverse | CCTATTTCTGCAGCATATTTGTAATCAG |  |  |
| ICV | ICV-set1 | Probe | ROX-ACCCAGACGACTACACACCAGAC  ATCCG-BHQ2 | M | This study |
|  |  | Forward | CTGGAGACTTYTTGGGAGTGGAGT |  |  |
|  |  | Reverse | TCTCAACCAAGCTGTGATTGTTCC |  |  |
|  | ICV-set2 | Probe | ROX-ACCCAGACGACTACACACCAGAC  ATCCG-BHQ2 |  |  |
|  |  | Forward | TTRGGAGTGGAGTCRATGATGAAA |  |  |
|  |  | Reverse | AACCAAGCTGTGATTGTTCCTATT |  |  |
|  | ICV-set3 | Probe | ROX-ACTGGCCTTGGAGAAGAAGCACT  ATC-BHQ2 |  |  |
|  |  | Forward | GAAGTAAGAAAAGCCAGCACAGC |  |  |
|  |  | Reverse | TTGCCATTCGTCCCATCTCTC |  |  |
|  | ICV-set4 | Probe | ROX-TGAAGGCAGAGTTGGAGAGATG  GGRCGA-BHQ2 |  |  |
|  |  | Forward | GACCCCACTTGGAAAAAGCAATA |  |  |
|  |  | Reverse | CAAGCAGAAGCAGGTTGACTTRT |  |  |
|  | ICV-set5 | Probe | ROX-TGAAGGCAGAGTTGGAGAGATG  GGRCGA-BHQ2 |  |  |
|  |  | Forward | AGACCCCACTTGGAAAAAGCAAT |  |  |
|  |  | Reverse | AGCAGAAGCAGGTTGACTTRTTAT |  |  |
| HBoV | HBoV-set1 | Probe | VIC-ACACCCATAAAGTCTAATCCCTT  GGACGA-BHQ1 | NS1&NP1 | This study |
|  |  | Forward | GGCCTGCCTCAACAGGTAAA |  |  |
|  |  | Reverse | AAGCACTCCTCCCACCAAAC |  |  |
|  | HBoV -set2 | Probe | VIC-AGCGAGAGGCATGGGGTGGAGTT-BHQ1 |  |  |
|  |  | Forward | CTCTTCTGGCTACACGTCCTT |  |  |
|  |  | Reverse | ATGAAGGTCTCCTCTGCGAT |  |  |
|  | HBoV-se3 | Probe | VIC-TTTCCCCGATGTACTCTCCCTCGT  CTT-BHQ1 |  |  |
|  |  | Forward | TCGAAGACCTCAGACCAAGTGAT |  |  |
|  |  | Reverse | GCGAGTAGAGTGCCAGTAGAAC |  |  |
|  | HBoV-set4 | Probe | VIC-TTTCCCCGATGTACTCTCCCTCGT  CTT-BHQ1 |  |  |
|  |  | Forward | AAGACCTCAGACCAAGTGAT |  |  |
|  |  | Reverse | GGAAGCTCTGTGTTGACTGAA |  |  |
|  | HBoV-set5 | Probe | VIC-TCAGACTGCATCCGGTCTC-BHQ1 |  |  |
|  |  | Forward | CCTATATAAGCCGATGCACTTC |  |  |
|  |  | Reverse | GCTTTTTCCAGAGATGTTCAC |  |  |
| HAdV | HAdV-set1 | Probe | FAM-TGCAGTTNGCCCGCGCMACCGA-BHQ2 | Hexon | This study |
|  |  | Forward | ATGCACATCKCGGGMCAGGACG |  |  |
|  |  | Reverse | CCACCGTGGGGTTTCTAAACTT |  |  |
|  | HAdV-set2 | Probe | FAM-CTGGTGCAGTTCGCCCGTGCCA-BHQ2 |  | (Sanghavi et al., 2011) |
|  |  | Forward | GGACGCCTCGGAGTACCTGAG |  |  |
|  |  | Reverse | ACGTGGGGTTTCTGAACTTGTT |  |  |
|  | HAdV-set3 | Probe | FAM-TGCAGTTNGCCCGCGCMACCGA-BHQ2 |  | This study |
|  |  | Forward | CAGGACGCYTCGGAGTACC |  |  |
|  |  | Reverse | GCCACCGTGGGGTTTCTAA |  |  |
|  | HAdV-set4 | Probe | FAM-TGCAGTTNGCCCGCGCMACCGA-BHQ2 |  | This study |
|  |  | Forward | TGCCGCAGTGGTCTTACAT |  |  |
|  |  | Reverse | ACCGTGGGGTTTCTAAACT |  |  |
|  | HAdV-set5 | Probe | FAM-TGCAGTTNGCCCGCGCMACCGA-BHQ2 |  | This study |
|  |  | Forward | GGACGCCTCGGAGTACCTGAG |  |  |
|  |  | Reverse | ACGTGGGGTTTCTGAACTTGTT |  |  |
|  | HAdV-set6 | Probe | FAM-ATYGGCTCKCATCCTCGCAC-BHQ2+TMP |  | This study |
|  |  | Forward | CCAAAGGMCCCCATCCARGT |  |  |
|  |  | Reverse | CTACTTYCAYCACATCAACAG |  |  |
|  | HAdV-set7 | Probe | FAM-ATYGGCTCKCATCCTCGCAC-3'MGB+TMP |  | This study |
|  |  | Forward | CCAAAGGMCCCCATCCARGT |  |  |
|  |  | Reverse | CTACTTYCAYCACATCAACAG |  |  |
| HCoV-OC43 | HCoV-OC43-set1 | Probe | FAM-CTGGCATGGACACCGCATTGTT  GAG-BHQ1 | N&M | This study |
|  |  | Forward | AATTACCGACTGCCATCAACC |  |  |
|  |  | Reverse | TGCTTACCAGGAGTAAAAGACATC |  |  |
|  | HCoV-OC43-set2 | Probe | FAM-CTGGCATGGACACCGCATTGTT  GAG-BHQ1 |  |  |
|  |  | Forward | ACTGCCATCAACCCAAAAGG |  |  |
|  |  | Reverse | ACGCTCTACTACTGGATTGC |  |  |
|  | HCoV-OC43-set3 | Probe | FAM-TRCCAGGCGGAAACCTAGTCGG  AAT-BHQ1 |  |  |
|  |  | Forward | TCAATACCCCGGCTGACAT |  |  |
|  |  | Reverse | CCTGAGCCTTCAATATAGTAACCC |  |  |
|  | HCoV-OC43-set4 | Probe | FAM-AGGCTCTGCTGGATGTGCGCGA  AGT-BHQ1 |  |  |
|  |  | Forward | CAGGAAGGTCTGCTCCTAATTCCA |  |  |
|  |  | Reverse | AGAATTGGCTCTACTACGCGAT |  |  |
| HCoV-229E | HCoV-229E-set1 | Probe | AAGAACCTGACTCCCGTGCTCCTTCCC | N | This study |
|  |  | Forward | VIC-GCTCCCAAATGGTGTTACTGTT-BHQ1 |  |  |
|  |  | Reverse | AGGATTCCGAGATTGAGRTTTGGA |  |  |
|  | HCoV-229E-set2 | Probe | VIC-AGTCCTGCTTCTTCTCAATCTGCT  GCC-BHQ1 |  |  |
|  |  | Forward | GTACCCCTAAGCCTTCTCGTAA |  |  |
|  |  | Reverse | TTAGGCTGTCTTTTCCACCGT |  |  |
|  | HCoV-229E-set3 | Probe | VIC-AGTCCTGCTTCTTCTCAATCTGCT  GCC-BHQ1 |  |  |
|  |  | Forward | GTACCCCTAAGCCTTCTCGTAA |  |  |
|  |  | Reverse | ATCGTTAGGCTGTCTTTTCCAC |  |  |
|  | HCoV-229E-set4 | Probe | VIC-AGTCCTGCTTCTTCTCAATCTGCT  GCC-BHQ1 |  |  |
|  |  | Forward | GTACCCCTAAGCCTTCTCGTAA |  |  |
|  |  | Reverse | AACTCTGAGAACGAGCAAGAA |  |  |
|  | HCoV-229E-set5 | Probe | VIC-AGTCCTGCTTCTTCTCAATCTGCT  GCC-BHQ1 |  |  |
|  |  | Forward | GTACCCCTAAGCCTTCTCGTAA |  |  |
|  |  | Reverse | AGAACTCTGAGAACGAGCAAG |  |  |
|  | HCoV-229E-set6 | Probe | VIC-TCACACAATGTTTTGGCCCCAGAG  ACC-BHQ1 |  |  |
|  |  | Forward | AGACAGCCTAACGATGATGTGAC |  |  |
|  |  | Reverse | CACAACACCTGCACTTCCAA |  |  |
| EV | EV-set1 | Probe | ROX-TCCTCCGGCCCCTGAATGC-BHQ2 | 5’UTR | This study |
|  |  | Forward | CATGGTGCGAAGAGTCGATTGA |  |  |
|  |  | Reverse | CACCCAAAGTAGTCGGTTCCGC |  |  |
|  | EV-set2 | Probe | ROX-TCCTCCGGCCCCTGAATGC-BHQ2 |  |  |
|  |  | Forward | CATGGTGCGAAGAGTCGATTGA |  |  |
|  |  | Reverse | AGTAGTCGGTTCCGCYRCAGA |  |  |
|  | EV-set3 | Probe | ROX-CTACTTTGGGTGTCCGTGTTTC-BHQ2 |  |  |
|  |  | Forward | GCAASTCTGCRGCGGAACC |  |  |
|  |  | Reverse | ATTGTCACCATAAGCAGCCA |  |  |
| HCoV-HKU1 | HCoV-HKU1-set1 | Probe | ROX-ATGCCCCTCTACTGGTCAAGCGA  TGG-BHQ1 | ORF 1ab | This study |
|  |  | Forward | GCGGAGGAGTTGGCTAGT |  |  |
|  |  | Reverse | AATGCAGCGACAATCTATCTTCA |  |  |
|  | HCoV-HKU1-set2 | Probe | ROX-ATGCCCCTCTACTGGTCAAGCGA  TGG-BHQ1 |  |  |
|  |  | Forward | CAGCGGAGGAGTTGGCTA |  |  |
|  |  | Reverse | GTCCAAGAATGCAGCGACAAT |  |  |
|  | HCoV-HKU1-set3 | Probe | ROX-ACTAGCCAACTCCTCCGCTGCA  TCC-BHQ1 |  |  |
|  |  | Forward | CCAGAATTTCGTTGGCTGCT |  |  |
|  |  | Reverse | AACACTTTCCATCGCTTGACC |  |  |
|  | HCoV-HKU1-set4 | Probe | ROX-ACTAGCCAACTCCTCCGCTGCA  TCC-BHQ1 |  |  |
|  |  | Forward | ATACGGTCTCGGCTTCAAGT |  |  |
|  |  | Reverse | TAACCCACCCTCATCTGACTT |  |  |
|  | HCoV-HKU1-set5 | Probe | ROX-ACATACCCTTTAGGAAAACCACC  CAAGC-BHQ1 |  |  |
|  |  | Forward | AAACAGCCGTAAAGTCCGGTA |  |  |
|  |  | Reverse | GTATGAACGGAACAAGCCCAT |  |  |
| HCoV-NL63 | HCoV-NL63-set1 | Probe | FAM-CTTGTGCCCATGCTGCTGTTGATT  CCT-BHQ1 | ORF 1ab | This study |
|  |  | Forward | TAGTGGTAAGTCACATTGTTCCAT |  |  |
|  |  | Reverse | AGCTCTTGCAGGTATAATCCTAGT |  |  |
|  | HCoV-NL63-set2 | Probe | FAM-CTTGTGCCCATGCTGCTGTTGATT  CCT-BHQ1 |  |  |
|  |  | Forward | TAGTGGTAAGTCACATTGTTCCAT |  |  |
|  |  | Reverse | CAATGCTATAAACAGTCATAGCTT |  |  |
| HPIV-4 | HPIV-4-set1 | Probe | VIC- TTCTGGGATGAGTGGTGYTCTGR  CTGT -BHQ1 | HN | This study |
|  |  | Forward | AGATCACAATGAACTACWGATGCC |  |  |
|  |  | Reverse | AYTCTGATGGGTGGAKTCTGGTG |  |  |
|  | HPIV-4-set2 | Probe | VIC-TTCTGGGATGAGTGGTGYTCTGRC  TGT-BHQ1 | N |  |
|  |  | Forward | AACTACWGATGCCAGAGCTTGA |  |  |
|  |  | Reverse | GTTGGAKTCTGGTGCGAGAT |  |  |
|  | HPIV-4-set3 | Probe | VIC-CAGGGGCAAGACTCTCSAACTAY  GAGC-BHQ1 |  |  |
|  |  | Forward | CAATGTCTCTRAACAGATCAGCTT |  |  |
|  |  | Reverse | TGCATTGTAGATTCTGGAWAGGC |  |  |
|  | HPIV-4-set4 | Probe | VIC-CAGGTACRCTGCTTATGGGAYCA  GACACAC -BHQ1 |  |  |
|  |  | Forward | TCTCCATGATATCCAACARACCC |  |  |
|  |  | Reverse | CAAGAATGAGTCCTTGATAGGTC |  |  |
|  | HPIV-4-set5 | Probe | VIC-TCACAGCCAGAGCACCACTCATC  CCA-BHQ1 |  |  |
|  |  | Forward | TCTCCATGATATCCAACARACCC |  |  |
|  |  | Reverse | CAAGAATGAGTCCTTGATAGGTC |  |  |
| HPIV-1 | HPIV-1-set1 | Probe | FAM-TCGCATATCTGAGCCAATTCCTGT  TTG-BHQ1 | HN | This study |
|  |  | Forward | CAAAGTTCAGTACAAAGCGGGAT |  |  |
|  |  | Reverse | CTGCATGGTGAATAGCAATRGTG |  |  |
|  | HPIV-1-set2 | Probe | FAM-TCGCATATCTGAGCCAATTCCTGT  TTG-BHQ1 |  |  |
|  |  | Forward | AGTTCAGTACAAAGCGGGATC |  |  |
|  |  | Reverse | CTGCATGGTGAATAGCAATRGTG |  |  |
|  | HPIV-1-set3 | Probe | FAM-TCGCATATCTGAGCCAATTCCTGT  TTG-BHQ1 |  |  |
|  |  | Forward | ACATACAAAGTTCAGTACAAAGCG |  |  |
|  |  | Reverse | CTGCATGGTGAATAGCAATRGTG |  |  |
|  | HPIV-1-set4 | Probe | FAM-ACCCCTCTGGACCCACATGATTTC  TGG-BHQ1 |  |  |
|  |  | Forward | AATTGGCTCAGATATGCGAGA |  |  |
|  |  | Reverse | GTTGTTGCTCARTAGGGGTTCTC |  |  |
|  | HPIV-1-set5 | Probe | FAM-AGTTATGCTCCTTGCCCACTGTGA  ATG-BHQ1 |  |  |
|  |  | Forward | CAACAGGAAATCATGTTCTGTAATAG |  |  |
|  |  | Reverse | CAAATACTAAGTCTTCTATACCTTCAC |  |  |
| HPIV-2 | HPIV-2-set1 | Probe | VIC-CCCTCAGCAACATCTCCCMAYGG  GTG-BHQ1 | HN | This study |
|  |  | Forward | GGACGCCTAAATATGGACCTCT |  |  |
|  |  | Reverse | TCGAGGCAATCTCCAAGTATTAC |  |  |
|  | HPIV-2-set2 | Probe | VIC-TCACTGCTATACCAGGAGGBTGTG  TCT-BHQ1 |  |  |
|  |  | Forward | ACCTAAGTGATGGAATCAATCGC |  |  |
|  |  | Reverse | AGCTAGRTCAGTCGTGGCATAAT |  |  |
|  | HPIV-2-set3 | Probe | VIC-TCACTGCTATACCAGGAGGBTGTG  TCT-BHQ1 |  |  |
|  |  | Forward | ACCTAAGTGATGGAATCAATCGC |  |  |
|  |  | Reverse | TCTCAGTTCAGCTAGRTCAGTCG |  |  |
|  | HPIV-2-set4 | Probe | VIC- AATCAATCGSAAAAGCTGTTCAG  TCAC -BHQ1 |  |  |
|  |  | Forward | GSRTTTCCAATCTTCAGGACTATG |  |  |
|  |  | Reverse | CAAGTCTCAGTTCAGCTAGRTC |  |  |
| HPIV-3 | HPIV-3-set1 | Probe | ROX-CCCTGGTCCAACAGAYGGGTATA  GTGC-BHQ2 | HN | This study |
|  |  | Forward | ATGATGGCTCAATCTCAACAAC |  |  |
|  |  | Reverse | TTCAAGACCTCCATACCCGAG |  |  |
|  | HPIV-3-set2 | Probe | ROX-CCCTGGTCCAACAGAYGGGTATA  GTGC-BHQ2 |  |  |
|  |  | Forward | TGGCTCAATCTCAACAACAAGA |  |  |
|  |  | Reverse | GACCTCCATACCCGAGAAATAT |  |  |
|  | HPIV-3-set3 | Probe | ROX-CCAGGTCACCCAGTTGTGTTGC  AGA-BHQ2 |  |  |
|  |  | Forward | TCTCGGGTATGGAGGTCTTGAACA |  |  |
|  |  | Reverse | ATGCYTGATTRCAGTCTCTCTGTGT |  |  |
|  | HPIV-3-set4 | Probe | ROX-CCGGGRCACCCAGTTGTGTTGC  AGA-BHQ2 |  |  |
|  |  | Forward | CCATCTGTTGGACCAGGGATA |  |  |
|  |  | Reverse | ATGCYTGATTRCAGTCTCTCTGTGT |  |  |
|  | HPIV-3-set5 | Probe | ROX-ATGCTTCCTGTGGGATTGAGTG  GAT-BHQ2 |  |  |
|  |  | Forward | CGGATGGATGTATAACGGGAGT |  |  |
|  |  | Reverse | GCTGTTGAGTAAGTTATGACTG |  |  |
| B2M | B2M-set1 | Probe | CY5-AGCAGGTTGCTCCACAGGT-BHQ2 | B2M | This study |
|  |  | Forward | TGCTGTCTCCATGTTTGATGTATCT |  |  |
|  |  | Reverse | TCTCTGCTCCCCACCTCTAAGT |  |  |

**Supplementary Table S2.** **The primers for *in vitro* transcription.**

| **Virus types** | **Primer names** | **Primer sequence (5'-3')** | **Product size (bp)** |
| --- | --- | --- | --- |
| IAV | Forward | ACGTTCTTTCTATCATCCCGTCAGG | 551 |
|  | Reverse | TAATACGACTCACTATAGGGAGCCATCTGTTCCATAGCCTT |  |
| IBV | Forward | GAATCCGTCTGCTGGAATTGAAGGG | 562 |
|  | Reverse | TAATACGACTCACTATAGGGCTCTTCTGGTGATAATCGGTGCTC |  |
| ICV | Forward | CAGAAGGAAGAGACATCAGAAGGCA | 532 |
|  | Reverse | TAATACGACTCACTATAGGGAGACAGGTCTTTAGATTGCAAGCAG |  |
| RSV | Forward | GGCTCTTAGCAAAGTCAAGTTGAATGA | 674 |
|  | Reverse | TAATACGACTCACTATAGGGGCTGGCACAGATGACTGGAAC |  |
| HRV | Forward | TAATACGACTCACTATAGGGGTGCCTACACAGAGCTTAGTAGGA | 556 |
|  | Reverse | CTTTAACTGGATCAGTGAATTTGCTTGGA |  |
| EV | Forward | TAATACGACTCACTATAGGGACTACTTCGGAAAACCTAGTAACACC | 557 |
|  | Reverse | GGCTGAATTTGAGTTTTCGTGAGAACCA |  |
| SARS-CoV-2 | Forward | TAATACGACTCACTATAGGGTCCCTATGGTGCTAACAAAGACGG | 639 |
|  | Reverse | ACTTATCGGCAATTTTGTTACCATCAGT |  |
| HCoV-HKU1 | Forward | TAATACGACTCACTATAGGGATACGGTCTCGGCTTCAAGT | 698 |
|  | Reverse | TACTGGTCTACAAAAAGTACGGGT |  |
| HCoV-229E | Forward | TAATACGACTCACTATAGGGGCTCCCAAATGGTGTTACTGTT | 764 |
|  | Reverse | ATAGAAACTTCATCACGCACTGG |  |
| HCoV-OC43 | Forward | TAATACGACTCACTATAGGGACTGCCATCAACCCAAAAGG | 747 |
|  | Reverse | AGAATTGGCTCTACTACGCGAT |  |
| HCoV-NL63 | Forward | TAATACGACTCACTATAGGGTAGTGGTAAGTCACATTGTTCCAT | 562 |
|  | Reverse | ACTAGCAGGTTTAACAGGGACAAA |  |
| HPIV-1 | Forward | ATACAAAGTTCAGTACAAAGCGGGAT | 792 |
|  | Reverse | TAATACGACTCACTATAGGGGCAAACACTCTGATTAACATTGGGACA |  |
| HPIV-2 | Forward | TGGGAGCATGTCCAACACCAAC | 633 |
|  | Reverse | TAATACGACTCACTATAGGGTGCTCTCACATATAAGGCAACCAT |  |
| HPIV-3 | Forward | AATAAGATTAATGCCGGGACCAGGA | 863 |
|  | Reverse | TAATACGACTCACTATAGGGAGTGTGTAATGCAGCTTGTTGTTGTA |  |
| HPIV-4 | Forward | TTGCAATGTCTCTAAACAGATCAGCTT | 568 |
|  | Reverse | TAATACGACTCACTATAGGGTCTTCTTTGCATCAAGAATGAGTCCT |  |
| HMPV | Forward | GTCTCTTCAAGGGATTCACCTGAGT | 807 |
|  | Reverse | TAATACGACTCACTATAGGGTTGTTAGATGACCTGGCAATGACCC |  |

T7 promoter sequence is underlined.

**Supplementary Table S3. Protocols for the nested RT-qPCR assay and Sanger sequencing.**

| **Target** | **Primer/Probe** | **Primer sequence (5'-3')** |
| --- | --- | --- |
| IAV | External forward primer | CAGGCCCCCTCAAAGCCGAG |
|  | External reverse primer | TCCATGTTGTTTGGGTCTCCATTTCCAT |
|  | Internal forward primer | GTCTTCTAACCGAGGTCGAA |
|  | Internal reverse primer | AGGTGACAGGATTGGTCTTG |
|  | Probe | VIC-TCAGGCCCCCTCAAAGCC-BHQ1 |
| HRV | External forward primer | GTGTTCTAGCCTGCGTGGCTGC |
|  | External forward primer | CATTTTGTTTAGATACCTGAGCGCCCATG |
|  | Internal forward primer | GACARGGTGKGAAGRKYC |
|  | Internal reverse primer | ACACGGACACCCAAAGTAGT |
|  | Probe | CCRCATTCAGGGGCCG |
| HMPV | Forward | ACAGTCAGAAGAGCTAACCG |
|  | Reverse | TGTTAGATGACCTGGCAATGAC |
| HBoV | Forward | GGAGATCATAAACACGCCCAGGAAGTGACG |
|  | Reverse | ACATAAGTGAAAGCAGGTTGAGAGAAAGCTCTAATTACAGG |
| HAdV | Forward | CCRAAGGMCCCCATCC |
|  | Reverse | GGGCACKTRGCGAC |
| HCoV-229E | Forward | GTACCCCTAAGCCTTCTCGTAA |
|  | Reverse | TGCACTTCCAAAGTTGTGGT |
| EV | Forward | TCCTCCGGCCCCTGAAT |
|  | Reverse | GGCTGAATTTGAGTTTTCGTGAGAACCA |
| HP1V-4 | Forward | ATCACAATGAACTACWGATGCC |
|  | Reverse | CAAGAATGAGTCCTTGATAGGTC |
| HP1V-1 | Forward | ACCCGAAATGAYAACTCCA |
|  | Reverse | GCATGGTGAATAGCAATRGT |
| HPIV-2 | Forward | TGTACACCCGGAAATCTGC |
|  | Reverse | TCGAGGCAATCTCCAAGTATTAC |
| HP1V-3 | Forward | CATCTGTTGGACCAGGGATA |
|  | Reverse | GCTGTTGAGTAAGTTATGACTG |

**Nested RT-qPCR reaction mixtures and thermal cycling parameters:** The reaction system includes 1× amplification buffer, 200μM dNTPs, 2U Taq DNA polymerase, 40U reverse transcriptase, 30U RNase inhibitor, 0.15 U UNG enzyme (Vazyme, China), and 5μL sample RNA. For each detection target, the final concentrations of the external primers, internal primers, and probes were 0.08μmol/ L, 0.4μmol/ L, and 0.2μmol/ L, respectively. Amplification was performed on an ABI 7500 real-time fluorescent quantitative PCR instrument using the following program: 55 ℃ for 15 min, followed by 95 ℃ for 1 min; pre-amplification for 15 cycles (95 ℃ for 10 s, 68 ℃ for 1 min); and then 40 cycles of detection amplification (95 ℃ for 10 s, 60 ℃ for 45 s). Fluorescent signals were collected during the annealing and extension phase at 60 ℃.

**Supplementary Table S4. Sequencing validation of the samples with inconsistent results by the new multiplex RT-qPCR assay and the commercial RT-qPCR kits.**

| **Sample No.** | **In-house assay** | **Commercial kit** | **Confirmation by Sanger sequencing** | |
| --- | --- | --- | --- | --- |
|  |  |  | **Sequence** (5’-3’) | **Virus** |
| S059 | EV | HRV | CACGTCCCACAGCCAGTGGGTAGTGTGTCGTAACGGGCAACTCTGCAGCGGAACCGACTACTTTGGGTGTCCGTGTTTCCTTTTATTCTTATGTTGGCTGCTTATGGTGACAATTAAAGAGTTGTTACCATATAGCTATTGGATTGGCCATCCGGTGTGCAACAGAGCGATCGTTTACCTATTTATTGGTTTTGTACCATTGACACTGAAGTCTGTGATCACCCTTAATTTTATCTTAACCCTCAACACAGCCAAACATGGGCTCACAGGTATCCACACAACGCTCTGGTTCTCACGAAAACTAAAATTCAGCCA | EVA71 |
| S066 | HPIV-1 | Negative | GGGTGCGCCGCAGGAAGGACACACATCTGGCTACTGATTACAACAGCAATGCATGCAACACTGTCCCTCATTATCATGATACTATGCATTGACCTAATTATGAAGCAAGACACTTGTATGAAGACAAACACCATGACAGTATCCTCCGTGAACGAAAGTGCCAAGACAATCAAAGAGACAATCACAGAATTAATCAGACAAGAAGTGATATCAAGGACTATAAACATACAAAGTTCAGTACAAAGCGGGATCCCAATATTGTTAAACAAGCAAAGCAGAGATCTCACACAATTAATAGAGAAGTCATGCAATAAACGGGAATTGGCTCAGATATGCGAGAACACCATTGCTATTCACCATGCA | HPIV-1 |
| S070 | HRV、EV | HRV | GGTGGCGTCGGACTTGCGACCCATGTTTAGCTGTGTTAAGGGTCAGGATATAATTAAGAGTGGTCACAGAATTCAGAGTTAATGGTACAAAACCAATAAATAGGTAAATAATTGCTCTGTTGCACACCGGATGGCCAATCCAATAGCTATATGGTAACAATTCTGCGATTGTCACCATAAGCAGCCAATATAAAGATAAAAGGAAACACGGACACCCAAAGTAGTCGGTTCCGCTGCAGAGTTACCCGTTACGACACACTACCCGCTGGCTTGTGGGCGTGTGCTCCGCAGTTAGGATTAGCCGCATTCAGGGGCCGGAGGAA | EVA71 |
| S082 | HRV、EV | HRV | TGGGGGGGAAAACGCCCACAGCCAGCGGGTAGTGTGTCGTAACGGGTAACTCTGCAGCGGAACCGACTACTTTGGGTGTCCGTGTTTCCTTTTATCTTTATATTGGCTGCTTATGGTGACAATCGCAGAATTGTTACCATATAGCTATTGGATTGGCCATCCGGTGTGCAACAGAGCAATTATTTACCTATTTATTGGTTTTGTACCATTAACTCTGAATTCTGTGACCACTCTTAATTATATCCTGACCCTTAACACAGCTAAACATGGGTTCGCAAGTGTCCGCACAGCGCTCTGGTTCTCACGAAAACTCAAATTCAGCCA | EVA71 |
| S084 | IAV、HAdV、HMPV | IAV | CCGGTTTGACTTCGCTGATTAGAATCATGGCATCGAGGATGAACTGCCTAATTACTGTTTTCCTCTGGATGGCATAGGACCAGGGCACAGGTATCAAGGCATTAAAGTTAAAACCGATGACGCTAATGGATGGGAAAAAGATGCTAATGTTGATACAGCTAATGAAATAGCCATAGGAAACAACCTGGCTATGGAAATTAATATCCAAGCTAACCTTTGGAGAAGTTTTCTGTACTCCAATGTGGCTTTGTACCTTCCAGATGTTTACAAGTACACGCCACCTAACATTACTTTGCCCACTAACACCAACACCTATGAGTACATGAACGGGCGAGTGGTATCCCCATCTCTGGTTGATTCATACATCAACATCGGCGCCAGGTGGTCTCTTGACCCAATGGACAATGTGAATCCATTCAACCACCACCGCAAACGCGGGA | HAdV-3 |
|  |  |  | GGAGTCAAAGATACCCTAGAATGGACATACCAAAAATTGCTAGATCCTTCTATGACTTATTTGAACAAAAAGTGTATCACAGAAGTTTGTTCATTGAGTATGGCAAAGCATTAGGCTCATCCTCTACAGGCAGCAAAGCAGAAAGTTTATTCGTCAACATATTCATGCAAGCTTATGGTGCTGGTCAAACAATGCTGAGGTGGGGGGTCATTGCCAGGTCATCTAACAA | HMPV- A |
| S109 | IAV、HPIV-1 | IAV | GGATTCCAATCTCGTAGTGTAGGATGGTCTTGGCTATTGATTGCAACAACAATGCATACAATATTGTCATTCATTATCATGATCTTATGCATTGACCTGATTATAAAACAAGACACTTGTATGAAAACGAATATCATGACAGTATCCTCCATGAACGAAAGTGCCAAAACAATCAAAGAGACAATCACAGAATTAATCAGACAAGAAGTAATATCAAGGACTATAAACATACAAAGCTCAGTACAAAGCGGGATCCCAATATTGTTAAATAAGCAAAGCAGAGATCTTACACAATTAATAGAGAAGTCATGCAACAGACAGGAATTGGCTCAGATATGCGAAAACACCATTGCTATTCACCATGCA | HPIV-1 |
| S116 | HAdV、HPIV-1 | HAdV | AATCAGGAGGACATACATCTGGCTACTGATTGCAACAACAATGCATGCAGTACTGTCCCTCATTATCATGATACTATGCATTGACCTAATTACAAAACAAGACACTTGTATAAAAACAAACATCATGACAGTATCCTCCGTGAACGAGAGTGCCAAAACAATCAAAGAGACAATCACAGAATTGATCAGACAAGAAGTGATATCAAGGACTATAAACATACAAAGTTCAGTACAAAGCGGGATCCCAATATTGTTAAACAAGCAAAGCAGAGATCTCACACAATTAATAGAGAAGTCATGCAACAAACAAGAATTGGCTCAGATATGCGAGAACACCATTGCTATTCACCATGCA | HPIV-1 |
| S172 | HRV、HAdV | HRV | TCCGAAGGTTCGCTATTGATCATGGCGTCGAGGATGAACTGCCTAATTACTGTTTTCCTCTGGATGGCATAGGACCAGGGAACAAATATCAAGGCATTAAACCTAGAGACACTGCATGGGAAAAAGATACTAAAGTTTCTACAGCTAATGAAATAGCCATAGGCAACAATCAGGCTATGGAAATTAATATCCAAGCTAATCTTTGGAGAAGTTTTCTGTACTCCAATGTGGCTTTGTACCTTCCAGATGTTTACAAGTACACGCCAACTAACATTACTCTGCCCGCTAACACCAACACCTATGAGTACATGAACGGGCGAGTGGTTTCCCCATCTCTGGTCGATTCATACATCAACATTGGCGCCAGGTGGTCTCTTGACCCAATGGACAATGTGAATCCATTTAACCACCACCGCAAACGCTGGA | HAdV |
| S173 | IAV、HAdV | IAV | GCCAAACCCAGTTCGCATTATAGAAATCATGGCATCGAGGATGAACTGCCTAATTACTGTTTTCCTCTGGATGGCATAGGACCAGGGCACAGGTATCAAGGCATTAAAGTTAAAACCGATGACGCTAATGGATGGGAAAAAGATGCTAATGTTGATACAGCTAATGAAATAGCCATAGGAAACAACCTGGCTATGGAAATTAATATCCAAGCTAACCTTTGGAGAAGTTTTCTGTACTCCAATGTGGCTTTGTACCTTCCAGATGTTTACAAGTACACGCCACCTAACATTACTTTGCCCACTAACACCAACACCTATGAGTACATGAACGGGCGAGTGGTATCCCCATCTCTGGTTGATTCATACATCAACATCGGCGCCAGGTGGTCTCTTGACCCAATGGACAATGTGAATCCATTCAACAACCACCGCAAACGCTGGA | HAdV-1 |
| S239 | HRV、EV | HRV | CGGGGGGACACGCCCACAGCCAGTGGGTAGTGTGTCGTAACGGGCAACTCTGCAGCGGAACCGACTACTTTGGGTGTCCGTGTTTCCTTTTATTCTTATGTTGGCTGCTTATGGTGACAATTAAAGAGTTGTTACCATATAGCTATTGGATTGGCCATCCGGTGTGCAACAGAGCGATCGTTTACCTATTTATTGGTTTTGTACCATTGACACTGAAGTCTGTGATCACCCTTAATTTTATCTTAACCCTCAACACAGCCAAACATGGGCTCACAGGTATCCACACAACGCTCTGGTTCTCACGAAAACTAAAATTCAGCCA | EVA71 |
| S248 | IAV、HPIV-1 | IAV | CATCTGGCCAGTCGGAGGACATACATCTGGCTACTGATTGCAACAACAATGCATGCAGCACTGTCCCTCATTATCATGATACTATGCATTGACCTAATTACAAAACAAGACACTTGTATAAAAACAAACATCATGACAGTATCCTCCGTGAACGAGAGTGCCAAAACAATCAAAGAGACAATCACAGAATTGATCAGACAAGAAGTGATATCAAGGACTATAAACATACAAAGTTCAGTACAAAGCGGGATCCCAATATTGTTAAACAAGCAAAGCAGAGATCTCACACAATTAATAGAGAAGTCATGCAACAAACAAGAATTGGCTCAGATATGCGAGAACACCATTGCTATTCACCATGCA | HPIV-1 |
| S286 | IAV、HPIV-3 | IAV | GGCATATATTTCTCGGGTATGGAGGTCTTGAACATCCAATAAATGAGAATGCAATCTGCAACACAACTGGGTGTCCCGGGAAAACGCAGAGAGACTGCAATCAGGCATCTCATAGTCCCTGGTTTTCAGACAGAAGGATGGTCAACTCCATTATTGTTGTTGACAAGGGCTTAAACTCAATTCCAAAACTGAAGGTATGGACGATATCCATGAGACAAAATTACTGGGGGTCAGAAGGAAGGCTACTTCTACTAGGTAACAAGATCTATATATATACAAGATCTACAAGTTGGCATAGCAAGTTACAATTAGGAATAATTGATATTACTGATTACAGTGATATAAGAATAAAATGGACATGGCATAATGTGTTATCAAGACCAGGAAACAATGAATGTCCATGGGGACATTCATGTCCAGATGGATGTATAACAGGAGTATATACTGATGCATATCCGCTCAATCCCACAGGGAGCATTGTGTCATCTGTCATATTAGACTCGCAAAAATCGAGAGTAAACCCAGTCATAACTCTCTTCAACCAGCACAA | HPIV-3 |
| S384 | HAdV、HPIV-4 | HAdV | GTTTTCACGGTATAAAACCTTCATCGTTCAGATTCACAGTCAGAGCACCACTCATTCCAGAAATCTCGCACCAGAATTCAAACACCAGAATGAGCAGAGATATCAACACAAGGGACAACACCAGAGCCGACCGTCAAAATACCAATGAGGATCGAGGCAGCAACATTCCAGATGACATCCTTGGAGATCTAGACAATTGACACATCAATAACCCAGCCATAGCTACACAACAATCATAAACCGGAAAAATCCCAAAGATACACAATCGCATACATACAATGAAAGCAATCACTCCTGTTCTATATTACAACCCAGAAATCCTACCAAACGCAACATCACGACCAATCAACTGATAATACATAGTAGAACACAAGAACACAACTGCATTGATTTAAGAAAAAACTAAGATAGGCCTGAACGGTTGCATTCAGGATTCTCAATCGTTCAGGCCAACACAATTCTCAGCTTCAAACGAAAACAACAAGATGAGCTTTGAAATAAGCATGGAGGAGATTGATGAGCTCATTGAAACTGGCAATCTCAACATAGATTATGCACTTAAAGAACTAGGTGCAGCCAGTCAATCCCCGCCAAACAAATCTCCGTCTCAGACCAGCAAAACAGAAGAAACCAACGATGATACAAGAACCTCAAGAAACTCGGCATCAGGAGAGGCACCAGCCCACACCTCTTCACTACTGCAGTCATACAATGAAGAGAGTGAATCAGAGAAACAGAGCTCAGACGGCTTCTCCATGATATCCAACAGACCTCAAACAGGTACACTGCTTATGGGATCAGACACACAATCTCCAAGTCCATCAAAGACCTATCAAGGCCTCCATTTCTTTGA | HPIV-4 |
| S397 | HPIV-2 | Negative | CCGTATCATGGATAACAAATTCCTTGTACTTAAATCATACAATGGGACGCCTAAATATGGACCTCTCCTAAATATTCCCAGCTTTATCCCCTCAGCAACATCTCCCAACGGGTGCACTAGAATACCATCATTTTCACTCATTAAGACCCATTGGTGTTACACTCACAATGTAATACTTGGAGATTGCCTCGAA | HPIV-2 |
| S399 | HPIV-3 | Negative | AAGCATATATTTTCTCCGGGTATGGAGGTCTTGACATCCAATAAATGAGAATGCAATCTGCAACACAACTGGGTGTCCTGGGAAAACACAGAGAGACTGTAATCAAGCATCTCATAGTCCATGGTTTTCAGATAGAAGGATGGTCAACTCCATAATTGTTGTTGACAAGGGCTTGAACTCAGTTCCAAAATTAAAGGTATGGACAATATCTATGAGACAAAATTACTGGGGGTCAGAAGGAAGATTACTTCTACTAGGTAACAAGATCTACATATACACAAGATCTACAAGTTGGCACAGCAAGTTACAATTAGGAATAATTGACATTACTGACTACAGTGATATAAGGATAAAATGGACATGGCATAATGTGCTATCAAGACCAGGAAACAATGAATGTCCATGGGGACATTCATGTCCGGATGGATGTATAACGGGAGTATATACCGATGCATATCCACTCAATCCCACAGGAAGCATTGTATCATCTGTCATATTGGACTCACAAAAATCGAGAGTCAACCCAGTCATAACTTCCTCCCAACAGCA | HPIV-3 |
| S476 | RSV、HAdV | RSV | CTAAGGTTCGGTAAATAGTAAATCATGGCGTCGAGGATGAACTGCCTAATTACTGTTTTCCTCTGGATGGCATAGGACCAGGGAACAAATATCAAGGCATTAAACCTAGAGACACTGCATGGGAAAAAGATACTAAAGTTTCTACAGCTAATGAAATAGCCATAGGCAACAATCAGGCTATGGAAATTAATATCCAAGCTAATCTTTGGAGAAGTTTTCTGTACTCCAATGTGGCTTTGTACCTTCCAGATGTTTACAAGTACACGCCAACTAACATTACTCTGCCCGCTAACACCAACACCTATGAGTACATGAACGGGCGAGTGGTTTCCCCATCTCTGGTCGATTCATACATCAACATTGGCGCCAGGTGGTCTCTTGACCCAATGGACAATGTGAATCCATTTAACCACCACCGCAAACGCTGGA | HAdV |
| S562 | IAV、HRV、HAdV | IAV、HRV | ACGGAGGTCGCTCATCGAATCATGGCATCGAGGATGAACTGCCTAATTACTGTTTTCCTCTGGATGGCATAGGACCAGGGCACAGGTATCAAGGCATTAAAGTTAAAACCGATGACGCTAATGGATGGGAAAAAGATGCTAATGTTGATACAGCTAATGAAATAGCCATAGGAAACAACCTGGCTATGGAAATTAATATCCAAGCTAACCTTTGGAGAAGTTTTCTGTACTCCAATGTGGCTTTGTACCTTCCAGATGTTTACAAGTACACGCCACCTAACATTACTTTGCCCACTAACACCAACACCTATGAGTACATGAACGGGCGAGTGGTATCCCCATCTCTGGTTGATTCATACATCAACATCGGCGCCAGGTGGTCTCTTGACCCAATGGACAATGTGAATCCATTCAACCACCACCGCAAAACGCGGGA | HAdV |
| S563 | IAV、  HCoV-229E | IAV | CACTCACTTCTGCCAAGAGTCTTGCTCGTTCTCAGAGTTCTGAAACAAAAGAACAAAAGCATGAAATGCAAAAGCCACGGTGGAAAAGACAGCCTAATGATGATGTGACATCTAATGTCACACAATGTTTTGGCCCCAGAGACCTTGACCACAACTTTGGAAGTGCAA | HCoV-229E |
| S579 | RSV、HBoV | RSV | TGCTCTTTCCAGAGATGTTCACTCGCCGGAGACCGGATGCAGTCTGATTGAATCAGGAAGTGCATCGGCTTATATAGGA | HBoV |
| S614 | IAV、HAdV | IAV | CGCGAATCCATTCGCATTATGAATCATGGCATCGAGGATGAACTGCCTAATTACTGTTTTCCTCTGGATGGCATAGGACCAGGGCACAGGTATCAAGGCATTAAAGTTAAAACCGATGACGCTAATGGATGGGAAAAAGATGCTAATGTTGATACAGCTAATGAAATAGCCATAGGAAACAACCTGGCTATGGAAATTAATATCCAAGCTAACCTTTGGAGAAGTTTTCTGTACTCCAATGTGGCTTTGTACCTTCCAGATGTTTACAAGTACACGCCACCTAACATTACTTTGCCCACTAACACCAACACCTATGAGTACATGAACGGGCGAGTGGTATCCCCATCTCTGGTTGATTCATACATCAACATCGGCGCCAGGTGGTCTCTTGACCCAATGGACAATGTGAATCCATTCAACAACAACCGCAAACGCTGGA | HAdV |

Consistent pathogens identified by both assays were not subjected to further confirmation by Sanger sequencing.

**Supplementary Table S5. GenBank accession numbers of enterovirus genomic sequences used in in-silico cross-reactivity analysis to confirm the specificity of the assay to HRV.**

| **Different categories of viruses** | CVA6 | CVA16 | EVA71 | EVD68 | echovirus 30 |
| --- | --- | --- | --- | --- | --- |
| **GenBank No.** | MN032612.1 | MT212036.1 | MW655540.1 | OR460083.1 | PV821997.1 |
|  | PP191126.1 | OQ091670.1 | LC626901.1 | PP548248.1 | OQ842411.1 |
|  | OQ026340.1 | LC506462.1 | OQ026338.1 | NC_038308.1 | PQ612485.1 |
|  | KX372340.1 | - | PQ868600.1 | PQ596456.1 | - |

**Supplementary Table S6. Feature comparison of the in-house assay with the commercial kits.**

| **Features** | **In-house assay** | **Sansure - 6-Plex Respiratory Pathogen Nucleic Acid Detection Kit** | **EasyDiagnosis - 2019-nCoV Nucleic Acid Detection Kit** |
| --- | --- | --- | --- |
| **Targets** | 18 respiratory viruses: IAV, IBV, ICV, RSV, HRV, EV, HAdV, SARS-CoV-2, HCoV-HKU1, HCoV-229E, HCoV-NL63, HCoV-OC43, HMPV, HBoV, HPIV-1/2/3/4 | 6 pathogens: IAV, IBV, RSV, HAdV, HRV, and *Mycoplasma pneumoniae* | SARS-CoV-2 |
| **Detection Time**  (excluding sample pretreatment) | ~90 minutes | ~100 minutes | ~75 minutes |
| **Cost** * (each sample) | ~3.4 $ | ~10.0 $ | ~0.65 $ |
| **Limit of Detection (LoD)** | 5-75 copies/25μL reaction system (equal to 100-1500 copies/mL | IAV and IBV: 2.0 TCID₅₀/mL;  RSV, HAdV, HRV, and *Mycoplasma pneumoniae*: 500 copies/mL | 500 copies/mL |
| **Cross-reactivity between HRV and EVA71** | Low | High | NA |

* Estimated cost for the in-house assay and average market price for the commercial kits.

NA, not applicable.


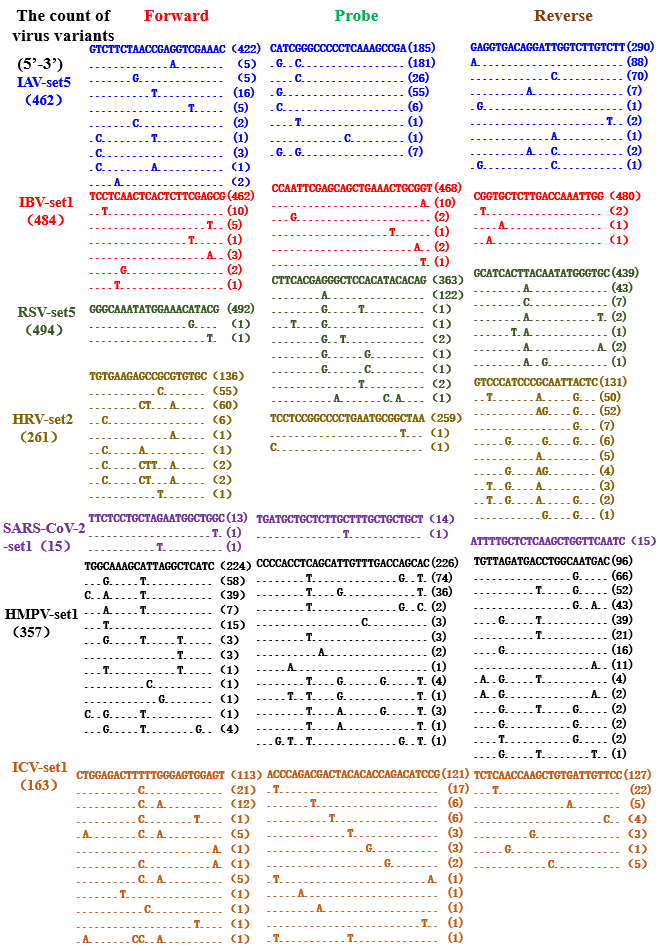


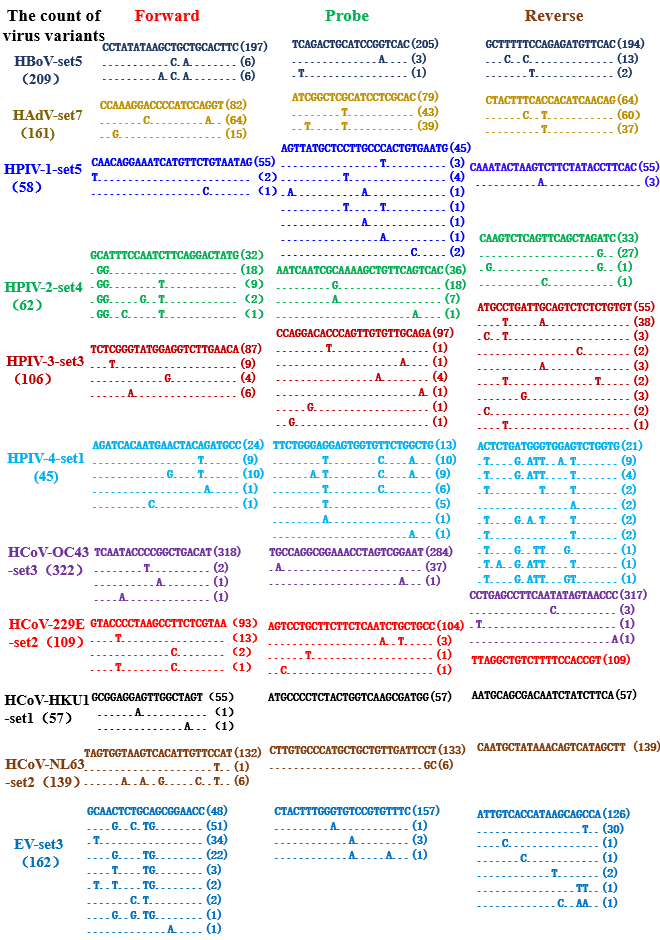


**Supplementary Figure S1. Sequence analyses of designed primers and probes.**


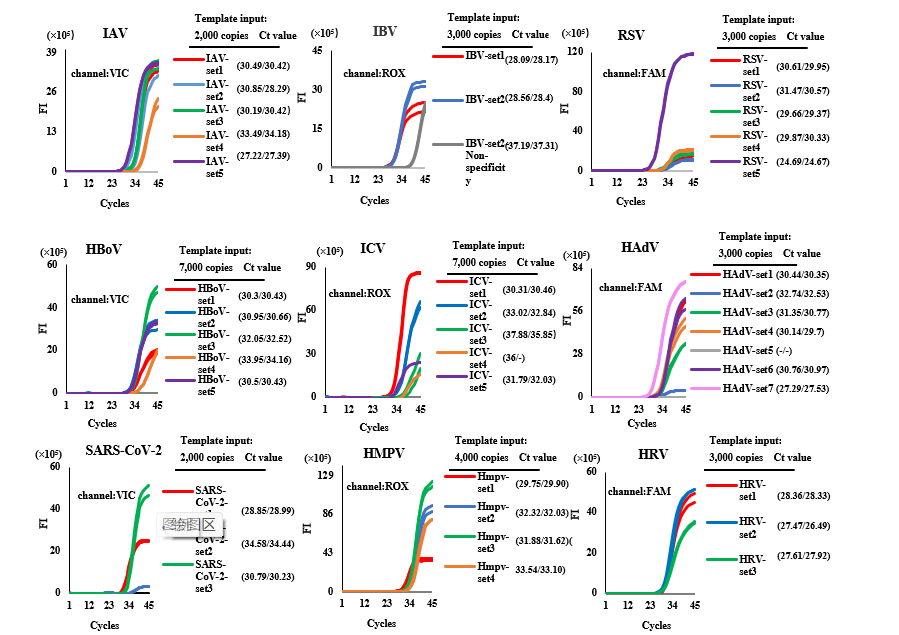


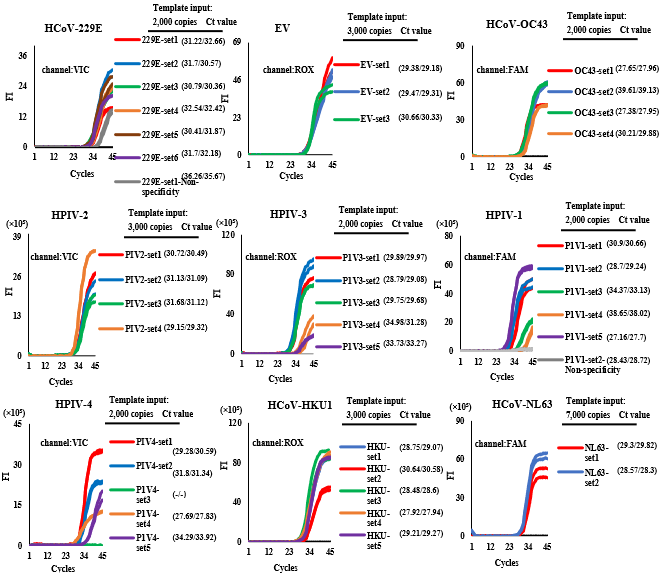


**Supplementary Figure S2. Optimization of the primers and probes for the multiplex RT-PCR assay.** *FI*, fluorescence intensity.


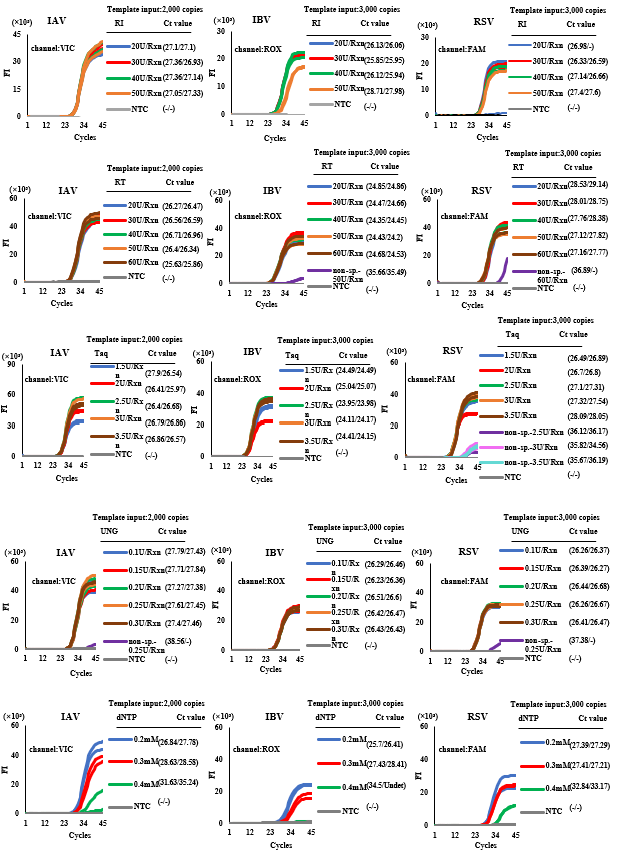


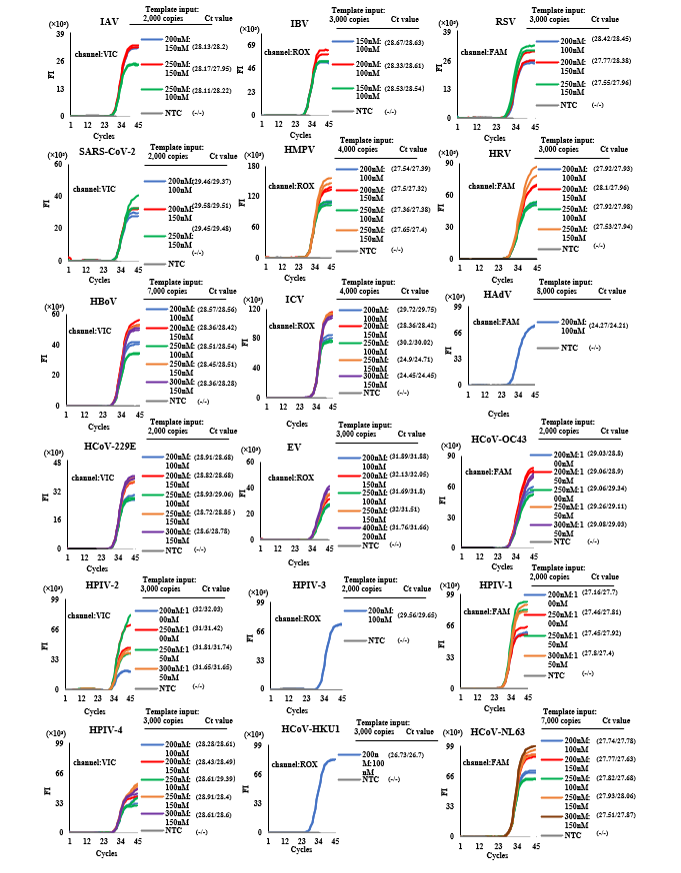


**Supplementary Figure S3. Optimization of the reaction condition of the multiplex RT-PCR assay.** *FI*, fluorescence intensity.


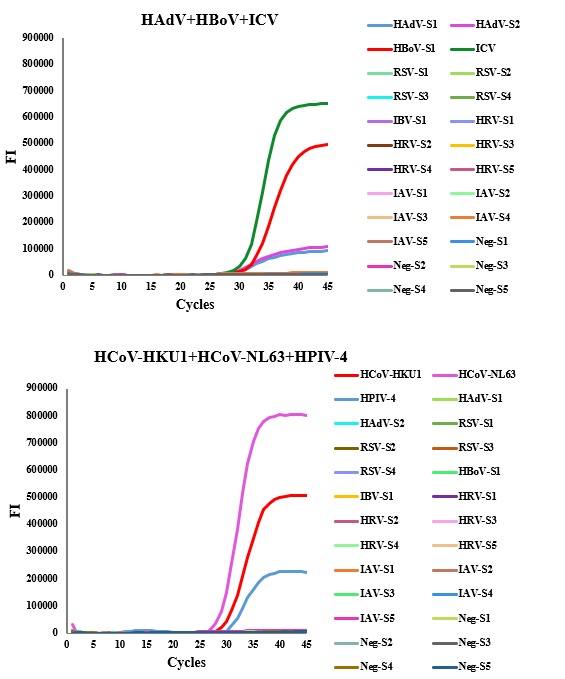


**Supplementary Figure S4.** **Specificity verification of the multiplex RT-qPCR assay.** *FI*, fluorescence intensity.


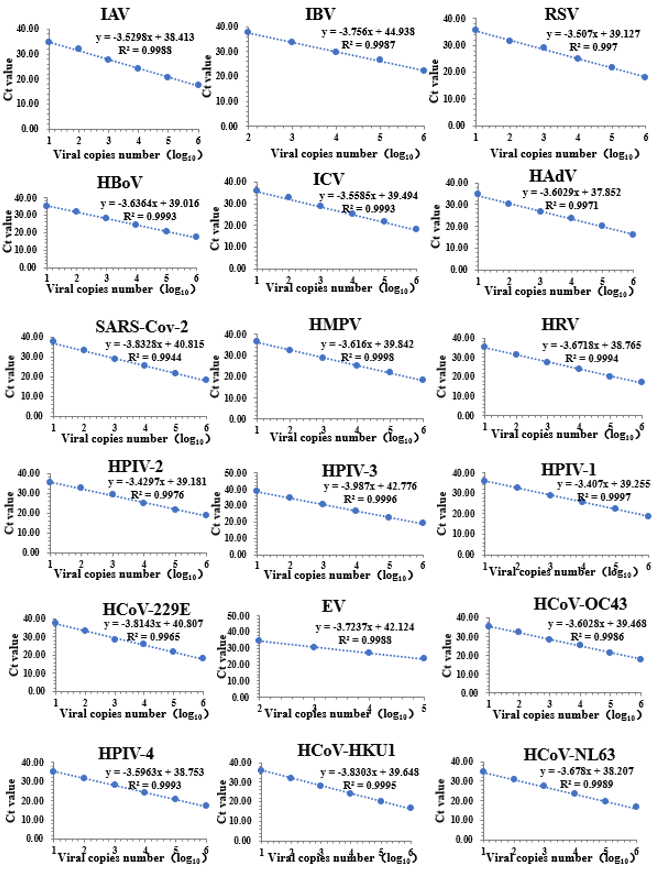


**Supplementary Figure S5.** **Standard curves of the singleplex RT-qPCR assays.**

**References:**

Feng, Z.-s., Zhao, L., Wang, J., Qiu, F.-z., Zhao, M.-c., Wang, L., et al. (2018). A multiplex one-tube nested real time RT-PCR assay for simultaneous detection of respiratory syncytial virus, human rhinovirus and human metapneumovirus. *Virology Journal* 15(1). doi: 10.1186/s12985-018-1061-0.

Frediansyah, A., Tiwari, R., Sharun, K., Dhama, K., and Harapan, H. (2021). Antivirals for COVID-19: A critical review. *Clinical Epidemiology and Global Health* 9**,** 90-98. doi: 10.1016/j.cegh.2020.07.006.

Lee, J.S., Ahn, J.J., Kim, S.J., Yu, S.Y., Koh, E.J., Kim, S.H., et al. (2021). POCT Detection of 14 Respiratory Viruses Using Multiplex RT-PCR. *BioChip Journal* 15(4)**,** 371-380. doi: 10.1007/s13206-021-00037-w.

Mancini, F., Barbanti, F., Scaturro, M., Fontana, S., Di Martino, A., Marsili, G., et al. (2021). Multiplex Real-Time Reverse-Transcription Polymerase Chain Reaction Assays for Diagnostic Testing of Severe Acute Respiratory Syndrome Coronavirus 2 and Seasonal Influenza Viruses: A Challenge of the Phase 3 Pandemic Setting. *The Journal of Infectious Diseases* 223(5)**,** 765-774. doi: 10.1093/infdis/jiaa658.

Sanghavi, S.K., Bullotta, A., Husain, S., and Rinaldo, C.R. (2011). Clinical evaluation of multiplex real‐time PCR panels for rapid detection of respiratory viral infections. *Journal of Medical Virology* 84(1)**,** 162-169. doi: 10.1002/jmv.22186.
